# A patient-specific therapeutic approach for tumour cell population extinction and drug toxicity reduction using control systems-based dose-profile design

**DOI:** 10.1186/1742-4682-10-68

**Published:** 2013-12-26

**Authors:** Suhela Kapoor, VP Subramanyam Rallabandi, Chandrashekhar Sakode, Radhakant Padhi, Prasun K Roy

**Affiliations:** 1 National Brain Research Centre, Manesar, Haryana 122050, India; 2 Indian Institute of Science, Bangalore, Karnataka 560012, India

**Keywords:** Tumour cell extinction, Cancer therapy optimization, Control system, Chemotherapy, Immunotherapy, Glioma, Astrocytoma, Meningioma, Oligodendroglioma, Glioblastoma

## Abstract

**Background:**

When anti-tumour therapy is administered to a tumour-host environment, an asymptotic tapering extremity of the tumour cell distribution is noticed. This extremity harbors a small number of residual tumour cells that later lead to secondary malignances. Thus, a method is needed that would enable the malignant population to be completely eliminated within a desired time-frame, negating the possibility of recurrence and drug-induced toxicity.

**Methods:**

In this study, we delineate a computational procedure using the inverse input-reconstruction approach to calculate the unknown drug stimulus input, when one desires a known output tissue-response (full tumour cell elimination, no excess toxicity). The asymptotic extremity is taken care of using a bias shift of tumour-cell distribution and guided control of drug administration, with toxicity limits enforced, during mutually-synchronized chemotherapy (as Temozolomide) and immunotherapy (Interleukin-2 and Cytotoxic T-lymphocyte).

**Results:**

Quantitative modeling is done using representative characteristics of rapidly and slowly-growing tumours. Both were fully eliminated within 2 months with checks for recurrence and toxicity over a two-year time-line. The dose-time profile of the therapeutic agents has similar features across tumours: biphasic (lymphocytes), monophasic (chemotherapy) and stationary (interleukin), with terminal pulses of the three agents together ensuring elimination of all malignant cells. The model is then justified with clinical case studies and animal models of different neurooncological tumours like glioma, meningioma and glioblastoma.

**Conclusion:**

The conflicting oncological objectives of tumour-cell extinction and host protection can be simultaneously accommodated using the techniques of drug input reconstruction by enforcing a bias shift and guided control over the drug dose-time profile. For translational applicability, the procedure can be adapted to accommodate varying patient parameters, and for corrective clinical monitoring, to implement full tumour extinction, while maintaining the health profile of the patient.

## Background

Though there has been considerable efforts in exploring newer modalities of cancer treatment in the last several decades, hopes have been belied by a fundamental reason, though not fully appreciated, namely the difficulty of eradicating a tumour mass due to the nature of reaction kinetics that govern the interaction of tumour cells with the therapeutic agents administered. These interactions are governed by first-order reaction processes (chemotherapy) and enzyme saturation kinetics (immunotherapy) [1]. Both these prescribed treatments cause an exponential decay of the tumour cell population leaving a finite definitive number of tumour cells at the asymptotic extremity of tumour versus drug distribution curve. By its very nature, an asymptotic tail implies the tumour cell population curve will contact the horizontal axis and become zero only when either the time duration or the drug dose is infinite. For instance, a typical tumour may have 10^10^ cells, and the surviving fraction, SF, of tumour cells after administration of a drug concentration D is SF = *exp* (−α D). Using the standard values of α = 0.02 and *D* = 150 mg for bleomycin (maximum dose tolerated) [2], the equation yields 1,765 cells surviving. This elucidates why many tumours recur, after appearing to initially shrink or regress under therapy.

An imminent question in neurooncology is the efficacy of the drug to be able to cross the “blood–brain barrier” (BBB) to enter the brain. A recent progress is the development of DNA alkylating drugs such as Temozolomide (TMZ) which while being highly targeted, can effectively cross the BBB [3]. However TMZ is known to be contraindicated in some cases such as in patients with severe myelosuppression. A few other common side effects are nausea and vomiting which are self-limiting or readily controlled with standard therapy. Temozolomide is a teratogenic compound and thus should not be used during pregnancy. It might very rarely leads to acute respiratory failure. However, no drug-related adverse CNS effects or alopecia are known to occur with temozolomide [3].

To complement the effects of chemotherapy, immunotherapy, is also being considered for its synergistic carcinolytic effects. Recent incisive findings of Wheeler *et al*. [4] indicate that a combinatorial therapy design utilizing immunotherapy with chemotherapy reduces tumour volume by 50% and appreciably extends the average 1 year survival duration of glioblastoma patients, which cannot be done by either chemotherapy or immunotherapy alone. An important aspect of immunotherapy is to utilize cytotoxic T-lymphocytes (CTL) which are CD8 + T cells, that are known to be carcinolytic. Activated CTL can be generated by administering immunomodulating factors,. This can be done by two means: (i) administering cytokines such as Interleukin-2 (IL-2) which can cross the blood–brain barrier, and (ii) injecting cellular agents such as tumour-infiltrating lymphocytes (TIL) prepared beforehand by sensitizing T-cells of the patient’s blood against the tumour (biopsy tissue). These TILs proceed satisfactorily to the tumour mass in the brain parenchyma. Also, IL-2 is well known to stimulate recruitment and proliferation of cytotoxic T-lymphocytes, such as CD8+ T cells, suggesting a novel neurooncological approach [5].

A critical aspect of anticancer therapy is maintaining protection of the healthy (nonmalignant) cells of the affected as well as neighboring unaffected tissue within tolerable limits. In cases of toxicity due to high doses of chemotherapeutic or immunotherapeutic agents, the patient may succumb to opportunistic pathogenic infection occurring due to the massive reduction of the normal protective immune cells by the agents, thus leading to septicemia, multi-organ failure and eventually death. The normal cell protection can be characterized by the concentration of circulating lymphocyte population in the blood [6]. These lymphocytes generate antibodies against infection, activate B-cells, T-cells and Natural Killer (NK) cells against pathogens. For ensuring that normal tissue protection is maintained during therapy, we have applied constraints or bounds in the feedback function of the drug dosages in the therapy control system, so that there is always a self-regulated judicious combination of the dose-and-time schedule of the antitumour agents, such that the circulating lymphocytes do not fall below a tolerable threshold value. The therapeutic agents have compensatory effects on the lymphocytes, as the chemotherapy input damages the lymphocytes, while the immunotherapy drug protects the lymphocytes. A measure of the tissue damage inflicted by antitumour agents can be described by the toxicity cost function *J*, wherein cytotoxicity, a second-order effect [7], depends on second-power of drug-dosage:

J=12B1u12+B2u22+….,

Here *u*_*1*_*, u*_*2*_*,* …. are the levels of the different antitumour agents, while *B*_*1*_*, B*_*2*_*,* …. are the weighting factors of the different agents. We use this principle to suitably orchestrate the temporal schedule of the drugs, so that that toxicity is minimized.

We may mention that various attempts at modeling the immune system interaction with neoplastic tumours have been previously made [8-10]. These models have efficiently characterized the computational dynamics of drug versus tumour interaction via the immune system. Using the background of the existing models, in our model we have tried to delineate the kinetics and dynamics of immune modulation responsible for the paradoxical clinical phenomenon of tumour dormancy, prolonged arrest and oscillations of tumour-size [11]. A unitary approach to the dual behaviour of tumour progression and tumour regression has recently been explained [12], where the neoplastic process has been elucidated as systems biology-based abnormality. The tumour regression approach that we report in the present work is to our knowledge, the first endeavor to elucidate a quantitative methodology to delineate the dose-time profile of administration of the antitumour agents (chemotherapy, interleukin, lymphocytes) with neuroncological cases as examples, so as to enforce the tumour cell population to zero, thus enabling full tumour elimination. For this, we develop an interdisciplinary approach, utilizing input reconstruction analysis and bias shift.

## Methods

### Inverse construction of drug input for obtaining desired tumour response

In conventional quantitative mathematical models, the inputs in terms of therapeutic agent concentration are substituted in the model (differential equations of tumour cell population) to solve for tumour cell population at different dosages and time durations. This is a “forward direct solution”, whereby, given the stimulus or input (concentrations of the *N* number of drugs, *D*_*N*_), one calculates the response or output (remaining tumour cell population *T*), by considering the successive intermediary stages of cell-drug interactions (X, Y, Z):

(1) $$D_{N}\left(\mathrm{drugs}\right)\to X\to Y\to Z\to T\left(\mathrm{tumour}\right)$$

**Figure 1:**
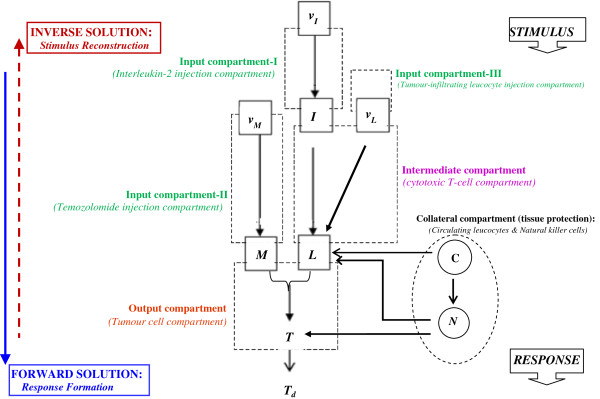
**Functional design of the therapeutic system using the Stimulus-Reconstruction approach.***Output compartment (Tumour cell compartment):* The state *T* (tumour cell population) is driven by state *L* (cytotoxic T-cell population in blood) and state *M* (chemotherapy drug temozolomide concentration in blood). Goal of system design is that the tumour cell population in this compartment decrease to a desired definitive value *T*_*d*_ (which is aimed to be zero); solving this compartment we arrive at L* and M* which are desired values respectively for cytotoxic T-cell population in blood and temozolomide concentration in blood required for complete tumour elimination. *Intermediate compartment (Cytotoxic T-cell compartment):* State *L* (cytotoxic T-cell population in blood) is driven by state *I* (interleukin-2 concentration in blood) and by state *v*_*L*_ (daily dosage of tumour-infiltrating leucocyte injection as immunotherapy). Solving this compartment, we get I* and *v*_L_* which are the desired values respectively of interleukin-2 concentration in blood and dosage of tumour-infiltrating leucocyte required for tumour elimination. *Input compartment-I (Interleukin-2 injection compartment):* State *I* (Interleukin-2 concentration in blood) is driven by state *v*_I_ (daily dosage of interleukin-2 injection as immunotherapy). By solving this compartment, we arrive at *v*_I_*, the desired value of interleukin-2 dosage required. *Input compartment-II (Temozolomide injection compartment):* State *M* (temozolomide concentration in blood) is driven by state *v*_M_ (daily dosage of temozolomide injection as chemotherapy). Solving this compartment, we arrive at *v*_M_*, the desired value of temozolomide dosage required. *Input compartment-III (Tumour-infiltrating leucocyte injection compartment):* This furnishes daily dosage of tumour infiltrating leucocyte injection required, and is constructed from cytotoxic T-cell compartment above. *Collateral compartment (normal tissue protection):* This compartment comprises of circulating lymphocytes concentration in blood (*C*) and natural killer cell concentration in blood (*N*) which guard normal tissue against infection that may be caused by toxicity side-effect induced by the therapeutic agents.

Typically, in eq. (1), the investigator starts with varying dosages of the drug combinations *D*_*N*_, and obtains the resulting values of the surviving tumour cell population *T* as one changes the dosage profiles of the drugs. Using this information, the specific dosing schedule of the drugs, *D*_*N*_^*†*^ is selected, which would lead to minimization of the number of remaining cancer cells, *T*_*min*_. The sites X, Y and Z are referred respectively as intrinsic (input), intermediate and extrinsic (output) compartments (Figure 1). The extrinsic compartment Z is related to the external output of the therapy system (i.e. remnant tumour cell population *T*). Likewise, the intrinsic compartment X relates to the internal control input coming from the therapy system, i.e., administration of therapeutic agents, *D*_*N*_.

To circumvent the problem of *T*_*min*_ i.e. the residual cancer cells that may cause tumour recurrence, we suggest the “backward inverse solution”, whereby one starts first with the desired objective, i.e. response or output (viz., tumour cell population becoming zero in a desired finite time period, *t*_*1*_), and, then work backwards through the sequential stages inversely, namely Z, Y, X to derive the drug concentrations to attain the above mentioned objectives:

(2) $$T\left(\mathrm{tumour}\right)\to Z\to Y\to X\to D_{N}\left(\mathrm{drugs}\right)$$

So, in the equation (2), one puts *T* = 0 at time *t*_*1*_, which is used to calculate the temporal profile of dosages of the combination drugs (D_N_^†^). Thus, if one administers the temporal dosages profile (D_N_^†^), then the tumour cell population definitively becomes zero in time *t*_*1*_, indicating tumour elimination. The perspective of inverse analysis approach [eq. (2)] has been well used in other fields in the form of stimulus reconstruction approach. This enables one to accurately quantify an antecedent unknown stimulus, using the knowledge of the observed response pattern. In our case, the methodology enables one to reconstruct the input drug stimulus, which will accurately produce complete tumour elimination as the output in finite time.

#### Problem of asymptotic drug reaction kinetics

**Figure 2:**
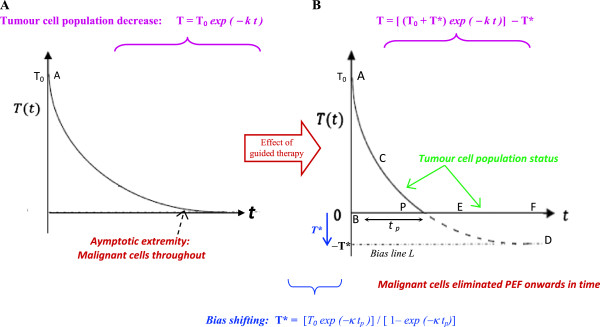
**Implementation of Bias Shift in tumour cell population trajectory to ensure complete elimination of tumour. (A)** Under usual intervention by therapeutic agents, the elimination of the tumour cell population *T(t)* is an exponentially decreasing curve, asymptotically approaching the time axis, so that tumour cell population becomes zero at infinite time duration. There is always a number of malignant cells in the exponential tail of the curve at any realistic time point, and the tumour recurs after the therapeutic agents are stopped. **(B)** The procedure from control systems analysis of utilizing the methodology of Bias Shift, for enabling the tumour cell population curve under therapy, so as to definitively attain zero cell population (tumour extinction) within a specific time *t*_*p*_. The curve of *T(t)* intersects the time axis i.e., *T(t)* = 0, at the definitive time point P, by incorporating bias shift using a finite T* value.

The cell-drug interactions are governed by first-order chemical reaction rate kinetics [1] using which the tumour cell population *T* at time *t* decreases exponentially in a monotonic manner:

(3) $$T=T_{0}\;\exp\left(-\kappa_{T}t\right),\;i.e.\;T\prime +\kappa_{T}T=0.$$

where *T*_*0*_ is initial population, *T′* is the time derivative of *T*, and *κ*_*T*_ is the drug’s cytotoxic rate constant (Figure 2A). As explained before, there persists asymptotically a residual tumour cell population, which now needs to be dealt with. Hence, one needs a procedure to ensure that the monotonically decreasing curve contacts the horizontal axis at a definite finite time point *t*_*p*_*.* In other words, the cell population curve should be dynamically modulated by therapy so that its trajectory is actively guided, enabling the curve to contact the horizontal axis at time *t*_*p*_.

As in the case of a biochemical reactor, conditions may arise where the synthesis rate *R* following first-order reaction, needs to be controlled and stopped at a definitive time [13]. To explore the possibility of inducing the reactor's processing to stop (i.e., *R* = 0), one may omit the input feed of reactants which causes the reaction to slow, but not become zero even after any length of time leaving a small asymptotic level of reaction rate *R* persisting at a time *t*, since first-order kinetics indicates *R′ +* κ *R* = 0.

The asymptoticity in both the above cases is due to the fact that both the dynamic equations are similar and first-order, viz. *y′ + k y* = 0. Here the y-axis denotes the system parameter, as reaction rate or altitude, while the x-axis denotes time. The equation implies that at infinite time, the curve meets the y-axis, if the coordinates at this meeting point is *t*^*†*^ and *y*^*†*^, then *y*^*†*^ = 0, as *t*^*†*^ = ∞. Nevertheless, in the exponential systems above, we elucidate that the systems dynamics can be halted in a finite time (i.e., *y* = 0, at specific time *t*_*p*_), if one implements the concept of an adjustable or tunable bias shift, denoted by y*. This enables the performance curve to approach a value *y* → y* (a proposed pre-determined negative value), as *t* → ∞ [Figure 2B]. This ensures that the curve trajectory intersects the time axis, and has an exact value *y* = 0 robustly, at a definite time *t*_*p*_ [point *P*, in Figure 2B]. We have earlier methodologically simulated and validated realistically the procedure of bias shift, while respecting the requisite bounds or constraints imposed on the system [14]; thereby the exponential curve was induced to become zero within specific time interval, the error in the validation process being within 2.5%.

### Using bias shift as baseline regulating principle

The aim of control systems design is to enable the tumour cell population (state *T*) to track and approach the T* value i.e. T → T*, where T* has a chosen bias shift whose value depends on the desired duration of tumour elimination, *t*_*p*_. From Figure 2B, the curve *T* is the arc path ACPD.

(4) $$T=\left[\left(T_{0}+T^{*}\right)\exp\;\left(-\kappa_{T}t\right)\;]-T^{*},\mathrm{whereby},\mathrm{bias}\;T^{*}=-[T_{0}\exp\;\left(-\kappa_{T}t_{p}\right)\;]/[1–\exp\;\left(-\kappa_{T}t_{p}\right)\right]$$

To enable the *T* curve to approach towards the T* value (bias shifted line *L* in Figure 2B), we use the guided tracking principle of control systems analysis [15]. Actually, the tumour cell population *T* follows the curve from point A to P in Figure 2B, but when the *T* trajectory reaches *P* (i.e., when population *T* attains zero value), the curve becomes horizontal, as all the tumour cells have become extinct, and thereafter, the trajectory will follow the axis-line PEF onwards, where *T* remains zero throughout as time elapses. The *T* population does not diminish further towards a negative value as that is a biologically impossible feat. Further, we can treat the benign state of the subject (without any tumour cells) as the target baseline condition, and hence the undesirable tumour cell population state *T* is considered as the tumour system deviation or error *E*_*T*_ from the benign condition. We define the error (Figure 2B) as *E*_*T*_ = (T – T*), and the error’s time derivative as *E*_*T′*_ = (*T′* – T*′). Thus eq. (3) becomes the error-equation which is calculated as:

(5) $$E_{T}\prime +\kappa_{T}E_{T}=0,\;\mathrm{implying}\;\left(T\prime –T^{*}\prime \right)+\kappa_{T}\;\left(T–T^{*}\right)=0$$

Eq. (5) is derived by substituting the expressions of *E*_*T*_ and *E*_*T′*_ above, in the error-equation. In eq. (5), the term T*′, i.e. the temporal rate of change of T*, is zero, since value of T* is a pre-fixed constant (as decided by the experimenter). Thus, dropping the term T*′ in eq. (5) furnishes the required tumour cell reduction rate for tumour elimination:

(6) $$T\prime =-\kappa_{T}\left(T–T^{*}\right)$$

Eq. (6) is the condition for complete tumour regression, and hence should be followed by the tumour cell compartment in Figure 1. Thereafter, proceeding upwards through the successive compartments, such as administration of chemotherapy then the immunotherapy, we can obtain the dose-time profiles of temozolomide, interleukin-2 and tumour-infiltrating lymphocyte injections, for regression of the tumour within the time duration *t*_*d*_. Thereafter, the tumour shall not recur as there are no surviving tumour cells. As a precaution, we continue the therapies for an extra sufficiently long period before fully stopping.

We have earlier used the inverse solution approach, but without bias shifting, for two treatment scenarios: (i) controlling chemotherapy infusion (imatinib) in myeloid leukemia [16], and (ii) regulating therapeutic infusion for treatment of ionic metabolic or hypocalceamic imbalance (control target error < 5%) [17]. In these cases the tumour cell population regressed and the blood ionic level tended to approach the desired value with high stability and asymptotically, but complete tumour elimination could not be obtained at a definitive time. In the present paper, we have remedied this problem by using adjustable bias shifting.

### Computational model of multimodal therapy

**Figure 3:**
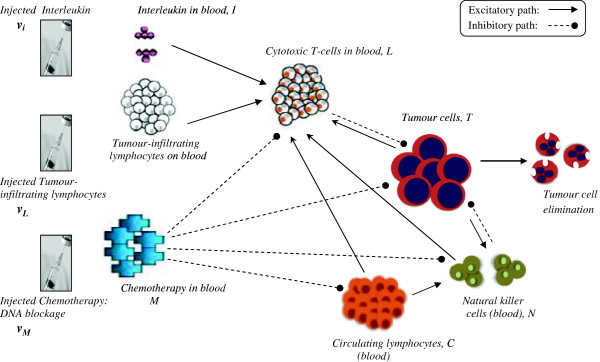
**Interaction of multimodal therapy on the tumour and host system.** Pointed and rounded arrows denote the major facilitatory and inhibitory interactions respectively. Chemotherapy inhibits the various cells, tumour cells being more susceptible. The major interactions are shown, and the minor ones are mentioned in the text.

We elucidate the quantitative approach for tumour dynamics under combinational chemotherapy and immunotherapy (Figure 3), based on coupled ordinary differential equations (ODE) between various cells and therapeutic agents. The approach is a broad one, and can be generalized to common situations. In the current instance, we take the case involving tumour cells, innate immunity cells (Natural killer cells), acquired immunity cells (Cytotoxic T-cells), and normal tissue protective cells (Circulating lymphocytes), along with antitumour therapies, including chemotherapy in the form of TMZ, and immunotherapy, namely externally administered lymphocytes (tumour-infiltrating lymphocytes) and lymphocyte-activating agents (as IL-2). The equations recollect the experimentally-based immunological reaction-rate framework demarcated by de Pillis *et al.*[18], Kuznetsov *et al.*[9] and Kirschner *et al.*[10], which has also been empirically validated [19]. The formulation also utilizes findings from animal studies and human clinical trials and uses models employed in cellular population biology, incorporating reaction kinetics such as Michaelis-Menten type of interaction and saturation processes as logistic functions.

**Figure 4:**
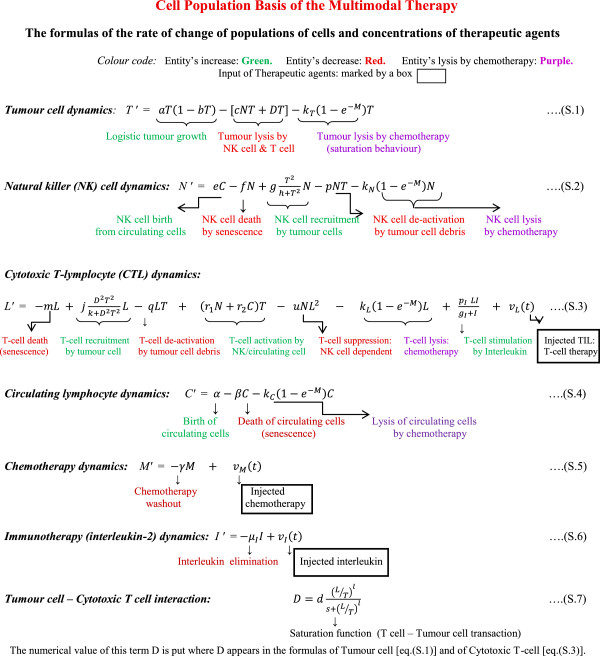
Quantitative Formulation of the Multimodal Therapy.

All the cell-cell interactions, rate equations with temporal dynamics of the proposed model are graphically summarized in Figure 4 [eq. (S.1) to eq. (S.7) therein], and these are elucidated in Additional file 1: Supporting Methods. In the formulas, the primed symbols denote the temporal derivatives of the above entities (populations of the four types of cells, or concentration of three therapeutic agents). The numerical values of the constant parameters are obtained from experimental or clinical studies; values are given in Table 1.

**Table 1:** Values of the biological and pathophysiological parameters for the tumour system

**Symbol**	**Numerical value**	**Characteristic significance**	**Reference**	**Units**
***Therapeutic parameters***				
*p*_I_	1.25 × 10^-1^	Interleukin 2-induced CD8+T-cell recruitment rate (maximum value)	Kirschner *et al.*[10]	per day (rate value)
*g*_I_	2.00 × 10^7^	Interleukin 2-induced CD8+T-cell recruitment (steepness value of the curve)	Kirschner *et al.*[10]	cell^2^ (cell-cell interaction)
μ_I_	1.0 × 10^1^	Decay rate of Interleukin-2 drug	Kirschner *et al.*[10]	per day
***Birth/death parameters***				
*α*	7.5 × 10^8^	Circulating lymphocyte birth rate (constant source)	de Pillis *et al.*[18]	cells per day
*β*	1.20 × 10^-2^	Lymphocyte death and differentiation rate	de Pillis *et al.*[18]	per day
*γ*	9.0 × 10^-1^	Chemotherapy drug decay rate	Calabresi *et al.*[20]	per day
*k*_*T*_	9.0 × 10^-1^	Chemotherapy-induced tumour cell lysis	Perry [1]	per day
*k*_*N*_*, k*_*L*_*, k*_*C*_	6.0 × 10^-1^	Chemotherapy-induced lysis of NK cells, CD8+T-cells, and Circulating lymphocytes, respectively	Perry [1]	per day
*r*_*1*_	1.1 × 10^-7^	CD8+T cell generation rate, induced by tumour cell lysis by NK cell	Yates *et al.*[21]; Lanzavecchia et al. [22]	per cell per day
*r*_*2*_	6.5 × 10^-11^	CD8+T cell generation rate, induced by tumour cell - circulating lymphocyte lysis	de Pillis *et al.*[18]	per cell per day
***Pathophysiological parameters***				
*a*	4.31 × 10^-1^ (high grade tumour); 3.01x10^-1^ (low grade tumour)	Rate of tumour growth	Calabresi *et al.*[20]; Stein [23]	per day
*b*	2.17 × 10^-8^ (high grade tumour); 1.02 × 10^-8^ (low grade tumour)	Deceleration effect of logistic growth of tumour	Calabresi *et al.*[20]; Stein [23]	per cell
*c*	6.41 × 10^-11^	NK cell-induced lysis of (non)-ligand-transduced tumour cell	Diefenbach *et al.*[24]	per cell per day
*d*	2.34	CD8+T cell-induced fractional tumour cell lysis (saturation value); priming by ligand-transduced cell	Dudley *et al.*[25]	per day
*e*	2.08 × 10^-3^	Lymphocyte fraction converting to NK cells	Kuznetsov *et al.*[9]	per day
*f*	4.12 × 10^-2^	NK cell death rate	Kuznetsov [9]	per day
*g*	4.98 × 10^-1^	Ligand-transduced tumour cell-induced NK cell recruitment rate (maximum value)	Dudley *et al.*[25]	per day
*h*	2.02 × 10^7^	NK cell recruitment by tumour cell (steepness index of recruitment curve)	Kuznetsov *et al.*[9]	cell^2^ (cell-cell interaction)
*l*	2.09	CD8+T cell-induced tumour cell lysis (exponent value)	Dudley *et al.*[25]	Dimensionless (exponent)
*s*	8.39 × 10^-2^	CD8+T cell-induced tumour cell lysis (Value of Steepness index of term D denoting lysis)	Dudley *et al.*[25]	Dimensionless (exponent)
*p*	3.42 × 10^-7^	Tumour-cell induced inactivation of NK cell	Diefenbach *et al.*[24]	per cell per day
*m*	2.04 × 10^-1^	CD8+T cell death rate	Yates *et al.*[21]	per day
*j*	2.49 × 10^-2^	Recruitment rate of CD8+T cells (max. value), cells primed with ligand-transduced tumour cells	Diefenbach *et al.*[24]	per day
*k*	3.66 × 10^7^	CD8+Tcell recruitment curve (steepness index), cells primed with ligand-transduced tumour cells	Diefenbach *et al.*[24]	cell^2^ (cell-cell interaction)
*q*	1.42 × 10^-6^	Tumour cell-induced CD8+T cell inactivation rate	Kuznetsov *et al.*[9]	per cell per day
*u*	3.00 × 10^-10^	CD8+T-cell regulation by NK-cell	de Pillis *et al.*[18]	per cell^2^ per day (cell-cell interaction rate)

#### Numerical values of the variables from the patient’s clinical data

Regarding the variables in the left side of eqs. (S.1)-(S.7), *N* is the NK cell population, *C* is circulating lymphocyte population, *L* is the cytotoxic T cell population in blood, *M* is the chemotherapeutic drug concentration (in serum), *I* is the immunotherapeutic drug concentration (in serum), and *T* is the tumour cell population in the tumour. One may note that all these variables have definitive numerical values, which vary as time elapses and therapy continues. These values can be calculated by solving the equations and using the numerical values of parameters in the left side of eqs. (S.1)-(S.6). Some of these parameters are related to the tumour cellular system:

(i) The pharmacological/cell birth/death/interaction parameters: *a, b, c, d, e, f, p, m, q, u, r*_*1*_*, r*_*2*_

(ii) Drug input/output parameters: α, β, γ, μ

(iii) Michaelis-Menten or saturation parameters: *g, h, j, k, p*_*I*_*, g*_*I*_*, D, d, l, s*;

(iv) Temporal lysis parameters of different cells: *k*_*T*_, *k*_*N*_, *k*_*L*_, *k*_*C*_.

The other parameters of the equations are related directly to the therapeutic agents:

(a) *v*_M_*(t), v*_I_*(t), v*_L_*(t)* are the daily injected dosages (input rate per day) of chemotherapy (temozolomide), immunotherapy (interleukin-2), and cytotherapy (TIL cells) respectively.

(b) *d*, the saturation level of fractional tumour cell kill by the cytotoxic T cells,

(c) *l*, the power-law exponent of fractional tumour cell kill by CD8+ T cells,

(d) *s*, the steepness coefficient of the cytotoxic T cell – tumour cell interaction curve

#### Stationary condition of the system in tumour elimination

The equilibrium points or stationary conditions of the system can be found by setting the derivatives of the variables to zero, namely *dT/dt =*0, *dN/dt =* 0, *dL/dt =* 0, *dC/dt =* 0, in eqs. (S.1)-(S.4) respectively. Solving for the case *t =* 0, the equilibrium points are as follows:

(6A) $$\left[T_{E},N_{E},L_{E},C_{E}\right]=\left[0,\mathrm{e\alpha}/\mathrm{\beta f},0,\alpha /\beta \right]$$

This equation implies that there is a definitive tumour-free stationary state. At this equilibrium point there is no presence of any malignant cell, i.e., the cytotoxic T-cell population *T*_E_ = 0, *L*_E_ = 0, while there are specific levels of the immune cells, NK cells and circulating lymphocytes, that correlate with the healthy state (*N*_E_ = *eα/βf*, *C*_E_ = *α/β*). Substituting the values of the parameters α*,* β*, e* and *f* from Table 1 and taking the average blood volume of 6 liters for an adult, we arrive at the natural killer cell equilibrium population = 430,000 cells for the whole subject and circulating lymphocytes population = 72 billion for the same. It is this equilibrium state that the system tends to settle to, in the occasion of successful therapy.

### System design formulation of multimodal therapy

In order to describe a system in terms of a control systems schema, we need to define the internal parameters (state variables characterizing the condition of the tumour system) and the external parameters (control parameters that can alter the tumour system). We formulate the ordinary differential equations (Figure 4), in terms of the control theory, whereby

(i) *System state variables* are the parameters indicating the internal biological condition of host-tumour interaction in the patient. The system state variables in this case are (1) Tumour cells (*T*), (2) Natural killer cells (*N*), (3) Circulating lymphocytes (*C*), (4) Cytotoxic T-Lymphocytes (*L*), as well as (5) blood concentrations of Interleukin (*I*) and of (6) temozolomide chemotherapy (*M*) [Figure 1]. The state variables are denoted as functions *X*_*n*_, where *n* = 1 to 6.

(ii) *Control variables of system* include the parameter values the external administrations of the three therapeutic agents, viz. the dose input rates of the injected chemotherapy (temozolomide) and immunomodulants (interleukin-2 and tumour-infiltrating lymphocytes), which are represented by *v*_M_*(t), v*_I_*(t),* and *v*_L_*(t)* respectively. These parameters vary with time and are measured in body-weight normalized rate units, e.g. in mg. of a drug per kg of body weight (or, per square metre of body surface) of the patient, given per day. These control variables are denoted as functions *U*_*m*_, where *m* = 1 to 3.

(iii) *Constraint conditions of system* indicate the quantitative requirements or thresholds that cannot be crossed application of the therapy. All the state variables need to be maintained within minimum and maximum values given in Table 2. For instance, the minimum and maximum values of the different cellular parameters should be within the physiological range that is necessary for homeostatic maintenance of the internal environment, or milieu interne of the patient. Too high a value of an entity can be toxic to the system, while, in some cases, there is a minimum value required so that the host can have proper immune surveillance to ward off foreign cells or microorganisms.

**Table 2:** Bound limits of biological and therapeutic parameters applicable to human cases

**Parameter**	**Lower limit**	**Upper limit**	**Reference**
Circulating Lymphocyte, C (total population in the individual)	2.32 × 10^9^ cells (for not more than 6 months duration)	4.5 × 10^11^ cells	Macintyre *et al.*[26] (upper limit); Jarosz *et al.*[27] (lower limit)
Natural Killer Cells, N (total population in the individual)	Negligible value (for not more than 3½ months duration)	5.85 × 10^10^ cells	Berrington *et al.*[28] (upper limit); Jawahar *et al.*[29] (lower limit)
Tumour-specific cytotoxic (CD8+) T-cells (CTL), *L* (total population of these cells in the individual)	Negligible value *	6.05 × 10^10^ cells	Dudley *et al.*[25] (upper limit); Roitt *et al.*[30] (lower limit)
Temozolomide infusion dosage rate, v_M_	0	200 mg/m^2^/day (4.45mg/kg/day)	Perry [1]
Interleukin-2 infusion dosage rate, *v*_*I*_	0	7.2 × 10^4^ I.U/kg/day	Perry [1]
Tumour Infiltrating lymphocyte (TIL) cumulative dosage (over full therapy duration, i.e., ∑ *v*_*L*_)	0	13.7 × 10^10^ cells	Dudley *et al.*[25]

* This is applicable to the time after the tumour has been eliminated and pertains to population of the effective CD8+ T-cells specifically active against the particular tumour-lesion in the body; after tumour elimination, there is no possibility of stimulation by tumour cells to initiate the generation of the active tumour-specific CTL. The various bound values in the Table can be relaxed by 10-15%, this relaxation in the limits of physiological parameters being accommodated by the homeostatic adaptation of the physiological system [31]. Note: For clarifications of values of the bounds, see endnote ^*a*^ before the references bibliography.

Using control systems practice, to construct the rate equation of any one particular state variable *X*_*p*_ (a biological parameter), we express its time derivative or rate parameter *X*_*p*_′, as a function of (i) the values of the various biological parameters that determine the system, namely *X*_*n*_, and (ii) the effect of the proximal causal control variables (drug factors, *U*_*m*_) on the biological variables (*X*_*n*_).

We shall now explain the construction of the control design, in terms of objective, operation and toxicity-minimization for each compartment.

#### Level 1: tumour cell compartment

##### Objective

The aim is to find the desired values, M* and L* of this compartment’s input parameters (Temozolomide blood level and Cytotoxic T-cell population), which will drive the compartment’s output (tumour cell population curve *T*) towards the line T*, until the *T* value becomes zero at point *P* in time *t*_*P*_ (Figure 2B).

##### Operation of the compartment

For the tumour cell compartment in Figure 1, we construct the rate equation of the output state variable, tumour cell population *T*, by expressing its time derivative *T'*, in terms of

(i) the function *f*_*T*_ (*X*_*n*_) which denotes the values of the various biological parameters that determine the tumour cell population system, namely the state parameters *X*_*n.*_ This explains the tumour cell alteration function due to two intrinsic biological (non-pharmacological) processes: (a) tumour cell growth term, due to logistic or saturable growth, (b) tumour cell elimination term due to natural killer cells.

(ii) the functions *g*_*T*1_ and *g*_*T*2_ that imply the biological effect of the compartment’s input therapy variables that show a dose–response saturation behavior *U*_*r*_ , which is produced by the two antitumour entities, cytotoxic T-cell population *L*, and temozolomide concentration in blood, *M*.

Thus,

(7) $$T\prime =f_{T}+\left[g_{T1}U_{M}+g_{T2}U_{L}\right]=f_{T}+b_{T}$$

where *U*_*M*_ and *U*_*L*_ indicate the anti-tumour behavior or therapeutic efficiency of Temozolomide and cytotoxic T-lymphocytes respectively, while *b*_*T*_ denotes the combined antitumour effect of these two therapeutic entities on tumour growth rate *T′* (Table 3). This antitumour behavior is quantitatively a saturation type of dose–response function (like Michaelis-Menten function, Hill function or similar activity function), which is a ubiquitous property of pharmacological responses. Thus Eq. (7) which is equivalent to eq. (S.1) of Figure 4, can be written in terms of Michaleis-Menten kinetics as,

(8) $$T\prime =\mathrm{aT}\left(1-\mathrm{bT}\right)-\mathrm{cNT}-\mathrm{DT}-\kappa_{T}\left(1-e{-}^{M}\right)T$$

**Table 3:** Description of therapy system terms

**Terms**	**Description**	**Reference formulation**
*U*_*M*_	Therapeutic efficiency factor of Chemotherapy	*U*_*M*_ = {1 − *exp*(−*M*)} [Eq. (10)]
*U*_*L*_	Therapeutic efficiency factor of Cytotoxic T-lymphocytes	*U*_*L*_ = *d L*^*l*^/(*s T*^*l*^ + *L*^*l*^) [Eq. (10)]
*U*_*I*_	Therapeutic efficiency factor of Interleukin-2	*U*_*I*_ = *p*_*I*_*LI*/(*g*_*I*_ + *I*) [Eq. (27)]
*b*_*T*_	Total tumour cell lysis effect from the two cytotoxic agents, Chemotherapy (temozolomide) and Cytotoxic T-cells	*b*_*T*_ = *U*_*M*_*g*_*T*1_ + *U*_*L*_*g*_*T*2_ [Eq. (12)]
*b*_*L*_	Total cytotoxic T-cell activation effect from the two Immuno-modulating agents, Interleukin and Tumour-infiltrating lymphocyte	*b*_*L*_ = *U*_*I*_ + *v*_*L*_ [Eq. (29)]

Comparing the right side of eq. (7) and (8),

(9) $$f_{T}=\;\left[\;\mathrm{aT}\left(1–\mathrm{bT}\right)–\mathrm{cNT}\right];\;g_{T1}=–k_{T}T,\;g_{T2=}–T$$

and

(10) $$U_{M}=\left[1–\exp\;\left(–M\right)\right];\;U_{L}=D=d\;L^{l}/\left(s\;T^{l}+L^{l}\right).$$

Now, for enforcing the tumour elimination using the tracking approach mentioned earlier, we use the previously described tumour growth rate as *T′* = −*κ*_*T*_ (T – T*). [One may take care to distinguish this kappa parameter, *κ*_*T,*_ the tumour decay rate (due to multimodal therapy), from the cytolysis rate parameter k_T_ of eq. (S.1) due to chemotherapy only]. Putting this expression of *T*′ = −*κ*_*T*_ (T – T*) in eq. (7), we obtain the condition that needs to be followed to induce complete tumour regression:

(11) $$f_{T}+g_{T1}U_{M}+g_{T2}U_{L}=–\kappa_{T}\left(T–T^{*}\right)$$

Rewriting eq. (11), we obtain the terms containing the pharmacological effect functions *g*_*T*1_ and *g*_*T*2_:

(12) $$g_{T1}U_{M}+g_{T2}U_{L}=b_{T}$$

where,

(13) $$b_{T}=–\kappa_{T}\left(T–T^{*}\right)–f_{T}=–\left[\kappa_{T}\left(T–T^{*}\right)+f_{T}\right]$$

Eq. (12) furnishes the relationship of the blood concentration of the antitumour agents (*U*_***M***_ and *U*_***L***_), which, if implemented, will ensure that the tumour cells undergo complete extinction.

Transposing the eq. (13),

(14) $$f_{T}+\kappa_{T}\left(T–T^{*}\right)+b_{T}=0$$

Eq. (13) implies that, for proper therapy, the combined therapy-induced anti-tumour effect term, *b*_*T*_ , should take care of two aspects: (i) the condition required for enforcing tumour cell population elimination, i.e. the expression -κ_*T*_ (T – T*), and (ii) the tumour cell growth due to biological processes, i.e. *f*_*T*_ (Table 3). At each time point, as tumour cell population changes, the cytotoxic therapy drive term *b*_*T*_ alters. Eq. (13) indicates that the relaxation decay term *b*_*T*_ has negative value if tumour is to regress (or *b*_*T*_ has value zero, if the tumour becomes arrested at a definitive volume and thereby becomes stable), thus here *b*_*T*_ *≤* 0. From eq. (12):

(14A) $$g_{T1}U_{M}\;+\;g_{T2}\;U_{L}-\;b_{T}=0$$

Eq. (12) furnishes the relationship of the blood concentration of the antitumour agents (*U*_***M***_ and *U*_***L***_), which, if implemented, will ensure the tumour cells undergo complete extinction.

##### Minimization of toxicity of antitumour therapy

However the blood concentrations *U*_***M***_ and *U*_***L***_ also needs to be regulated in such a way so that the combined toxicity effect on the patient is minimal. Hence we now need to minimize the normal tissue damage cost function due to the agents. Using the quadratic cost concept (see earlier “Background” section), we have the cost function for the tumour cell compartment as:

(15) JT=12rT1UM2+rT2UL2,

Here *r*_*T*1_ and *r*_*T*2_ are the weighting factors of the two therapeutic entities, temozolomide and cytotoxic T-cells. The cost function *J*_*T*_ needs to be minimized, and at the same time eq. (12) should be obeyed as a constraint. The minimization of the cost function, *J*_*T*_ = ½ [*r*_T1_ U_M_ ^2^ + *r*_T2_ U_L_ ^2^], is evidently a constrained optimization problem, and the optimality can be customarily solved by the method of Lagrange multiplier, λ. Thereby the augmented cost function *J* can be written as:

(16) J¯=12rT1UM2+rT2UL2+λgT1UM+gT2UL–bT

where the right sided expression within the second brackets {….} in eq. (16) incorporates the left side of the constraint equation [eq. 14(A)], as required by the Lagrange’s method. To pursue the minimization of the augmented cost function, we differentiate *J* with respect to the two variables U_M_ and U_L_. Thereby, we find out the minimization conditions, i.e., ∂*J/*∂*U*_*M*_ *=* 0, and ∂*J/*∂*U*_*L*_ *=* 0, whence we get the expressions for the two variables:

Temozolomide efficiency term,

(17) $$U_{M}=b_{T}g_{T1}/r_{1}G$$

Cytotoxic T-lymphocyte efficiency term,

(18) $$U_{L}=b_{T}g_{T2}/r_{T2}G$$

where,

(19) $$G=\left[\left({g_{T1}}^{2}/r_{T1}\right)+\left({g_{T2}}^{2}/r_{T2}\right)\right]$$

Further, from eq. (10):

(20) $$U_{M}=\left[1-\exp\;\left(-M\right)\right]\;\mathrm{and}\;U_{L}=d\;L^{l}/\left(s\;T^{l}+L^{l}\right)$$

##### Desired input values of the therapy levels

Solving the two equations of the last line, we obtain the desired values, M* (blood concentration of temozolomide) and L* (cytotoxic T-cell population):

(21) $$M^{*}=–ln\left(1–U_{M}\right),\;\mathrm{and}\;L^{*}={\left[s\;T^{l}U_{L}/\left(d–U_{L}\right)\right]}^{1/l}$$

In the last two equations, for the terms U_M_ and U_L_, we substitute their values from eq. (17) and (18) respectively. Thence, we arrive at the desired values of the temozolomide blood level and cytotoxic T-cell population, which, if implemented, will regress the tumour fully:

Desired temozolomide blood level:

(22) $$M^{*}=–ln\left[1–\left\{b_{T}{g_{T}}_{1}/{r_{T}}_{1}G\right\}\right],$$

Desired cytotoxic T-cell population:

(23) $$L^{*}=–{\left[sT^{l}\left(b_{T}{g_{T}}_{2}/{r_{T}}_{2}G\right)/\left\{d–\left(b_{T}{g_{T}}_{2}/{r_{T}}_{2}G\right)\right\}\right]}^{1/l}$$

#### Level 2: cytotoxic T-cell compartment

##### Objective

Here the goal is to find the desired values, I* and *v*_L_*, of this compartment’s input parameters (interleukin-2 blood level and tumour-infiltrating lymphocyte injection dose-rate), which would drive the compartments output, namely the cytotoxic T-cell population, *L* to its desired value L* mentioned in the last paragraph. This enforced driving needs to be faster than the earlier compartment (tumour cell compartment) and requires to be done till time *t*_r_, point *P*, when all the tumour cells have become eliminated (Figure 2B).

##### Operation of this compartment

We use the methodology of guided tracking principle of control systems analysis, which we have already used for the tumour cell population compartment [eq. (5)]. Likewise, we enforce the situation of the cytotoxic T-cell population *L* obeying the condition *E*_*L*_*′ + κ*_*L*_*E*_*L*_ = 0, where *κ*_*L*_ is a positive parameter and the deviate *E*_*L*_ = (L – L*). Using similar logic of the reduction of the deviate or error [eq. (6)], we obtain:

(24) $$L\prime =-\kappa_{L}\left(L–L^{*}\right)$$

We recollect the characteristic rate formulation of the tumour cell compartment, T′ = *f*_*T*_ (X_n_) + *b*_*T*_ [i.e. eq. (7)]. We now desire to formulate the characteristic rate formulation of the cytotoxic T-cell compartment in terms of L′. Thus, we express the temporal dynamics equation of L*′* [Eq. S.3 of Figure 4)] as:

(25) $$L\prime =f_{L}\left(X\right)+U_{I}+v_{L}$$

where *v*_*L*_ is the tumour-infiltrating lymphocyte injection dose-rate, *U*_I_ is the therapeutic efficiency relationship of interleukin-2, and *f(X*_n_*)* is a system function expressing the T-cell immunomodulation, viz. the biological and pharmacological variables, acting on this cytotoxic T-cell population. Compare eq. (25) with eq. (S.3) that states:

(26) $$\begin{array}{l} L'=\mathrm{dL}/\mathrm{dt}\\ \;=-\mathrm{mL}+\mathrm{Lj}\left(\left(D^{2}T^{2}\right)/\left(k+D^{2}T^{2}\right)\right)-\mathrm{qLT}\\ \;+\left(r_{1}N+r_{2}C\right)T-\mathrm{uN}L^{2}-Lk_{L}\left(1-e^{-M}\right)+p_{1}\mathrm{LI}/\left(g_{1}+I\right)+v_{L}\left(t\right) \end{array}$$

By this comparison, one notes that *U*_*I*_, the term dealing with interleukin level *I*, can be identified as:

(27) $$U_{I}=\left(p_{I}\mathrm{LI}\right)/\left(g_{I}+I\right)$$

and one can also express

(28) $$\begin{array}{ll} f_{L}\left(X\right)= & -\mathrm{mL}\\ & +jD^{2}{T^{2}}_{/}\left(\kappa +D^{2}T^{2}\right)L-\mathrm{qLT}\\ & +\left(r_{1}N+r_{2}C\right)T-\mathrm{uN}L^{2}–L\kappa_{L}\left(1-e^{-M}\right) \end{array}$$

Eliminating *L′* from eq. (24) and eq. (25), we note that

(29) $$U_{I}+v_{L}=b_{L}$$

Where

(30) $$b_{L}=-f_{L}\left(X\right)–\kappa_{L}\left(L–L^{*}\right)$$

Note here that *f*_*L*_*(X)* is the performance function of the cytotoxic T-cell compartment, a part of the the right-side of the eq. (26) except its last two terms, which are the therapy-input terms, dependent on the interleukin and tumour-infiltrating lymphocyte injected, the two terms being denoted as *b*_*L*_*,* In eq. (26), the last term *v*_L_ is the Tumour-infiltrating lymphocyte injection dose-rate, while the second-last term *[p*_*I*_*LI/(g*_*I*_*+I)]*, now denoted as *U*_I_, is the therapeutic efficiency relationship of interleukin-2. Evidently from eq. (26), the term *b*_*L*_ signifies the total cytotoxic T-cell activation effect by the two immunotherapeutic inputs: the interleukin efficiency term *U*_*I*_ and the tumour-infiltrating lymphocyte administration term *v*_L_ (Table 3). Indeed, the said equation indicates that the relaxation decay term *b*_*L*_ has positive value if the tumour regresses (or zero, if the tumour is arrested and thereby stable), i.e. *b*_*T*_ *≥ 0*. Actually, eq. (29) furnishes the relationship of the characteristics of the antitumour agents, interleukin and tumour infiltrating lymphocytes (U_I_ and *v*_L_), which if implemented, will ensure the full elimination of tumour.

##### Minimization of toxicity of antitumour therapy

In this compartment, the two antitumour agents are nterleukin-2 and Tumour-infiltrating lymphocytes. As explained for the earlier compartment, we need to minimize the toxicity cost function for the compartment where the two therapeutic input functions arriving at the compartment are Interleukin-2 (i.e., its efficiency function U_I_) and tumour-infiltrating lymphocyte (its injection dose-rate, *v*_*L*_). The cost function for this compartment is:

(31) $$J_{L}=1/2\;\left(r_{L1}{U_{I}}^{2}+r_{L2}{v_{L}}^{2}\right)$$

where *r*_*L*1_ and *r*_*L*2_ are the sensitivity weights due to the two aforesaid therapeutic moieties in this compartment. For minimization, the constraint requirement [*U*_*I*_ *+ v*_*L*_ = *b*_*L*_] from [eq. (29)] should be obeyed. Solving using the Lagrange multiplier method, we arrive at:.

Interleukin-2 efficiency term,

(32) $$U_{I}=b_{L}/{r_{L}}_{1}\;H$$

Tumour-infiltrating lymphocyte injection dose-rate,

(33) $$v_{L}=b_{L}/{r_{L}}_{2}\;H,$$

where

(34) $$H=\left[\left(1/{r_{L}}_{1}\right)+\left(1/{r_{L}}_{2}\right)\right]$$

##### Desired input values of the therapy level

By transposing *U*_*I*_ *=* {*(p*_*I*_*L*_*I*_*)/(g*_*I*_ *+ I)*} [i.e., eq. (27)], we obtain the desired value of I* (blood concentration of Interleukin-2):

(35) $$I^{*}=g_{I}U_{I}/\left(p_{I}L–U_{I}\right)$$

Substituting *U*_I_ from eq. (32) to eq. (35), we get the desired value of tumour-infiltrating lymphocyte injection dose-rate which will eliminate the tumour:

Desired interleukin-2 blood concentration,

(36) $$I^{*}=g_{I}b_{L}/\left(p_{I}L\;r_{L1}H–b_{L}\right)$$

#### Level 3: temozolomide injection compartment

##### Objective

Here the goal is to find the desired value, *v*_M_*, of this compartment’s input parameter (temozolomide injection dose-rate), which would drive the compartment’s output, namely the patient’s blood level of temozolomide *M* to its desired value M* as given in eq. (22). This driving needs to be done faster than the preceding compartment (T-cell compartment), and is to be done by time *t*_*p*_, point P (Figure 1).

##### Operation of this compartment

We need to implement the condition that *E*_*M*_*′ + κ*_*M*_*E*_*M*_ = 0, where the deviate *E*_*M*_ = (M – M*). Using similar reasoning as the earlier compartment, we get M*′* = −*κ*_*M*_ (M – M*). Eliminating *M′* from this equation and from eq. (S.5), we have:

(37) $$\mathrm{Desired}\;\mathrm{temozolomide}\;\mathrm{injection}\;\mathrm{dose}-\mathrm{rate}\;v_{M}*=\mathrm{\gamma M}–\kappa_{M}\left(M–M^{*}\right)$$

#### Level 4: interleukin-2 injection compartment

The condition necessary to be implemented is that *E*_*I*_*′ + κ*_*I*_*E*_*I*_ = 0 where the deviate *E*_*I*_ = (I – I*) Thus, *I′* = −*κ*_*I*_(I – I*). By eliminating *I′* using eq. (S.6), we note:

(38) $$\mathrm{Desired}\;\mathrm{interleukin}-2\;\mathrm{injection}\;\mathrm{dose}-\mathrm{rate},v_{M}*={µ}_{I}I–\kappa_{I}\left(I–I^{*}\right)$$

#### Level 5: tumour-infiltrating lymphocyte injection compartment

For this compartment, we can write from eq. (33) that:

(39) $$\mathrm{Desired}\;\mathrm{tumour}-\mathrm{infiltrating}\;\mathrm{lymphocyte}\;\mathrm{injection}\;\mathrm{dose}-\mathrm{rate},\;v_{L}*=b_{L}/{r_{L}}_{2}\;H$$

### Determining tumour regression rate constant, bias shift and therapeutic weights

#### Delineating the rate parameters κ_T_, κ_M_, κ_L_, κ_I_ and bias T*

These are rate constants of the tumour cell compartment, temozolomide compartment, cytotoxic T-cell compartment and interleukin compartment, respectively, as regression occurs under the action of multi-modal therapy. These are calculated from the desired rate of tumour regression, expressed as settling time *t*_s_ of the regression process (the time duration in which 90% of tumour has regressed), and is taken to be around 1–2 months. The tumour regression rate constant *κ*_*T*_ = 4/*t*_s_; so if *t*_s_ is 60 days, *κ*_T_ = 0.067 per day. On the other hand, the dynamics of the successively preceding compartments (e.g. chemo-therapy and cytotoxic T-cell compartments) need to be faster, as they causally influence the tumour cell compartment, and thus need to change more rapidly if they are to have a controlling influence on the tumour cell compartment (Figure 1). Thus, the time constants of the modular stages will be lesser, and hence the process rate constant will be higher. So, we can choose the rate constants *κ*_*L*_ and *κ*_*M*_ of cytotoxic T-cell compartment and temozolomide injection compartment respectively, such that they exceed *κ*_*T*_*.* Similarly, the rate constant *κ*_*I*_ of interleukin compartment (that causally acts on the T-cell compartment), is chosen to be higher that *κ*_*L*_.

Thus,

$$\left(\kappa_{L},\kappa_{M}\right)>\kappa_{T},\;\mathrm{and}\;\kappa_{I}>\kappa_{L}.$$

As explained earlier, in control analysis [15], a thumb rule used is: rate constants between two successive stages in a cascade control system are in the ratio of 1: 3. Thus, if we have the value of rate constant of the tumour cell population, κ_*T*_ and then we obtain the value of the other constants in terms of κ_*T*_ :

(40) $$\kappa_{L}=3\kappa_{T}\;;\;\kappa_{M}=3\kappa_{T};\;\kappa_{I}=3\kappa_{L},\;i.e.,\;\kappa_{I}=9\kappa_{T}$$

In the above example, since, *κ*_*T*_ = 0.067/day, we get *κ*_*L*_ = 0.201/day, *κ*_*M*_ = 0.201/day, and *κ*_*I*_ = 0.603/day. It may be noted that the tumour elimination time *t*_*p*_ (Figure 2) when 100% tumour has regressed, is longer than *t*_s_, and can be selected as 10 days more, i.e. *t*_*p*_ = 70 days. If the initial pre-therapy tumour cell population is estimated as *T*_*0*_ , then substituting the values of *T*_*0*_, *κ*_*T*_ and *t*_*p*_ in the right-sided expression in eq. (4), provides the value of bias shift T*.

#### Selection of therapeutic weights r_T1_ , r_T2_ and r_L1_ , r_L2_

These adaptable parameters, *r*_*T1*_*, r*_*T2*_*, r*_*L1*_*, r*_*L2*_ are needed for minimizing the toxicity cost of the therapy. It is these parameters that give a control to the investigator for maneuvering the tumour elimination process, under adjustable dosing of the drugs. Initially, the values of the tuning parameters *r*_*T1*_*, r*_*T2*_*, r*_*L1*_*, r*_*L2*_ which appear in derivation, are chosen by specific quantitative conditions (see Additional file 2: Supporting Analysis). To recapitulate, *r*_*T1*_ and *r*_*T2*_ are respectively the toxicity cost weighting factors of temozolomide and cytotoxic-T-cell, acting on the tumour cell population compartment, producing the cost *J*_T_ which is to be optimized.

For such optimization problems, it is known that the important characteristic to be considered is the ratio *r*_*T1*_*: r*_*T2*_[15]. Thus, we can take the parameter *r*_*T1*_ to have a normalized value of unity (i.e. *r*_*T1*_ = 1), thereby the task is to suitably choose or optimize the value of the other tuning parameter *r*_*T2*_. Correspondingly, *r*_*L1*_ and *r*_*L2*_ are respectively the toxicity cost weighting factors of interleukin-2 and tumour-infiltrating lymphocytes, which act on the cytotoxic T-cell compartment and produces the toxicity cost *J*_L_. Similarly, this cost can be minimized by normalizing *r*_*L1*_ = 1, and then optimizing *r*_*L1*_. All the tuning parameter values must be greater than or equal to zero. Using the upper and lower bounds that needs to be followed by the cellular and pharmacological variables (Table 2), we can calculate the numerical values of the therapeutic weight parameters (Additional file 2: item B.3 and Table 1 there).

### Efficiency of different combinations of therapeutic agents

During the analysis and modeling procedure, the control variables, i.e. parameters of the therapeutic agents, should have positive values and not attain negative nor imaginary values, even though the system dynamics [eq. (S.1-S.7)] has fractional exponent at some places [eq. (S.7)]. The situation is amended in case if any of the computed control variables, or of the three injected drug dosage rates calculated (*v*_M_*, *v*_I_* or *v*_L_*), becomes imaginary or negative. If this happens, the particular drug injection rate (say, for drug-A) is stopped and kept suspended (its dosage is henceforth taken to be zero), the simulation is continued with the presence of the other two drugs. As long as the dosage of the drug-A calculated remains negative or imaginary, drug-A is not injected, and the drug A’s value for the subsequent simulation steps becomes zero, while the other two drugs, whose calculated dosages continue to be positive numbers, are continued to be administered. Only when the computed dose of the drug-A turns positive, then the injection of drug-A is resumed according to that dose. To wit, the following alternative situations occur:

(i) The dosage parameters of all three drugs remain positive throughout the time duration: If so, all are given.

(ii) One drug becomes negative or imaginary for a particular time interval: this drug is stopped during that interval, and the other two drugs are continued as per the modeling procedure with the first omitted

(iii) Two drugs become negative or imaginary: here the remaining drug with a positive drug dosage rate is administered.

(iv) All the three drugs violate the positivity condition: then no drug is given, until one or more drug rates become positive at a subsequent time, upon which the administration of the drug/s is resumed.

**Figure 5:**
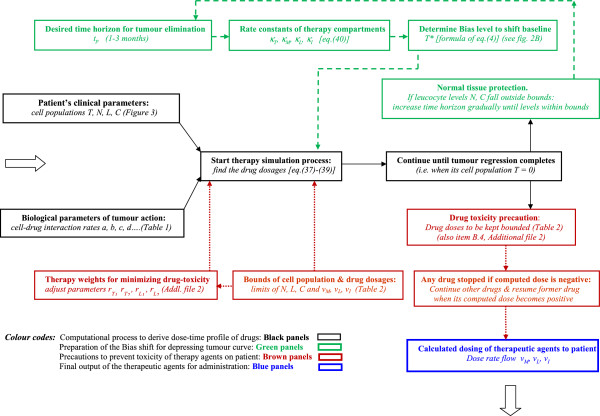
Flow sheet of multimodal therapy to actuate full elimination of tumour cells.

If case (ii) is applicable, then there will be three different combinations for two drugs at a time by permutation: (a) temozolomide and interleukin, or (ii) temozolomide and tumour infiltrating lymphocytes, or (iii) interleukin and tumour infiltrating lymphocytes. If case (iii) is relevant, then the three possibilities are: temozolomide or interleukin or tumour infiltrating lymphocytes. The mathematical formulation for cases (ii) and (iii) are derived later (Additional file 2: item B.4), using the overall approach of case (i) presented above. Further, once the tumour cell count becomes zero (point P, Figure 2B) by any of the above approaches, one stops the drug/s thereafter. The overall methodology of our approach is shown in Figure 5.

## Results and discussion

### Numerical simulations for tumour elimination

**Figure 6:**
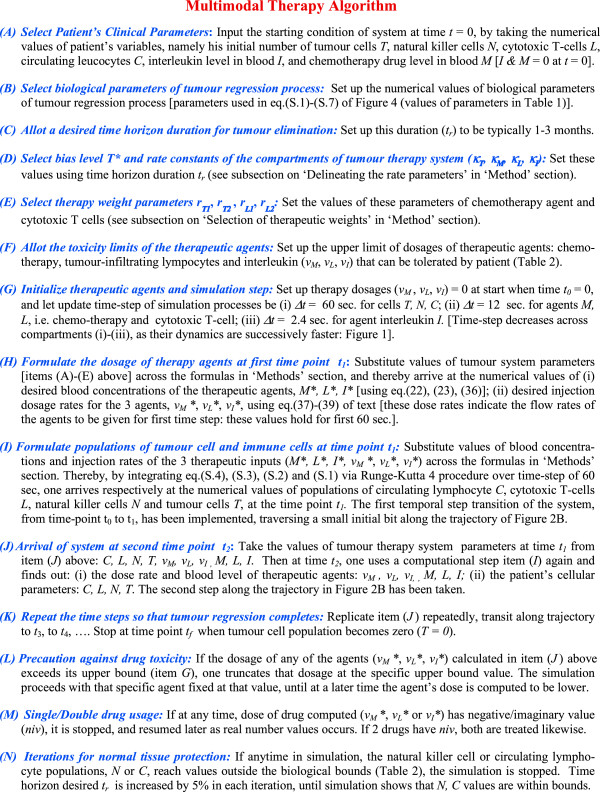
Steps of Multimodal Therapy Algorithm for Tumour Elimination.

We computationally model the tumour system dynamics using custom-made coding and solve in the MATLAB platform [eq. (7)-(14A)], and thereby obtain the temporal profile of the varying dose-rate of the therapeutic agents, by solving eq. (37)-(39) which respectively furnish the temporal profile of temozolomide, interleukin-2 and tumour-infiltrating lymphocytes, which would eliminate the tumour cell population. In Figure 6 we develop the detailed procedural steps into a readily-usable algorithm toolkit. Our concern is the general problem of neurooncology, especially neuroectoderm originating tumours such as glioma, melanoma or neuroblastoma, which are comparable tumours, biologically and pharmacologically, and respond to similar therapeutic interventions, as temozolomide, interleukin-2 and lymphocyte immunotherapy by T-cells [1,5,20,32,33]. Now we apply the proposed approach to regressing both slowly and rapidly growing tumours, astrocytoma grade II and astocytoma grade III respectively, aiming at a time-frame of about 2 months. We take the values of parameters of the equations from experimental clinical studies available or from general oncological investigations (Table 1). The values of the parameters can be applied to tumours in general, for example to tumours of neuroepithelial origin, as melanoma or neuroma, or sarcoma [9,19,24]. Actually, using values of such parameters from clinical or pre-clinical tumour model systems, one can solve differential equations of tumour response under drugs or immune cells, and the mathematical predictions are closely confirmed and validated by cytological measurements on human subjects during therapy [19,34,35].

#### Low-grade tumour

**Figure 7:**
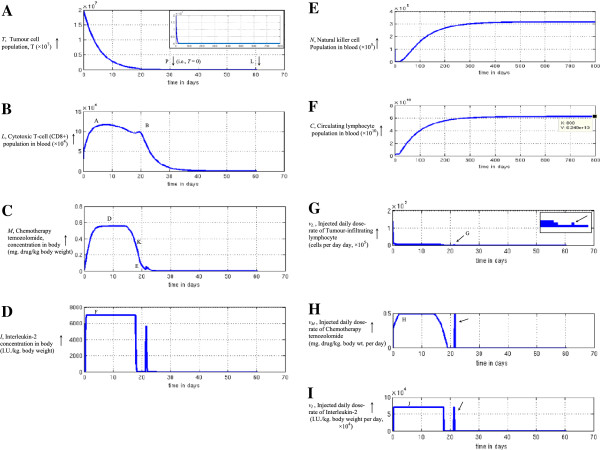
**Induction of complete elimination of low-grade tumour under multimodal therapy, inversely reconstructed with bias shift: oligodendroglioma tumour. (A)** Tumour cell population as therapy progresses, population becomes zero at point P, after 30.1 days, system tracked for 70 days (point L), to ensure complete elimination of the cells and no relapse of them occurs. Inset shows plot for 800 days, tumour cells population remains zero indefinitely. **(B)** Temporal variation of the Cytotoxic CD8+ lymphocyte population needed for complete elimination of tumour. Note bimodal temporal dose-profile with two peaks, occurring at two time points, A and B. **(C)** Temporal profile of concentration of chemotherapy agent Temozolomide required to eliminate the tumour. Observe unimodal temporal dose-profile with hump D, thereafter drug level decreases to E; **(D)** Level of Interleukin-2 concentration needed for tumour regression. Note the substantial level of interleukin, the level being truncated so as to keep concentration level below its toxicity threshold (point F level). **(E)** Natural killer cell population during therapy. Population stops increasing duly, 600 days plotted to show this and population does not cross upper bound of physiological limits in Table 2. **(F)** Circulating lymphocyte population, also does not exceed upper bound of physiological limits (Table 2). **(G)** Injected daily dose-rate of tumour-infiltrating lymphocyte required for tumour elimination. Arrow demarcates pulse dosage injection (point G) needed at latter portion of the therapy. Distinct step G readily seen in enlarged inset (arrow). **(H)** Injected daily dose-rate of Temozolomide chemotherapeutic drug necessary for tumour regression, dose-rate does not rise above H, so as to be below toxicity bound (Table 2). Arrow points to pulse dosage injection required later. **(I)** Injected daily dose-rate of Interleukin-2 required for eliminating the tumour, dose-rate also kept at permissible upper limit (Table 2). Arrow shows elevated pulse dosage injection needed later.

We consider the realistic case of low-grade tumour as ologidendroglioma or glioma grade II tumour with initial conditions of starting malignant cell population (*T*_*0*_) of 2 × 10^7^, natural killer cell population of 10^5^, cytotoxic T-cell population of 5 × 10^4^ and circulating lymphocytes of 10^9^ cells (see endnote ^*b*^ before the References). From this *T*_*0*_ value, we get a bias shift T* = 1.8986 × 10^5^ cells [right-side of eq. (4)] (see subsection on “Determining tumour regression rate constants” above). All the other constants used in the model are given in Table 1, with the tumour cell growth rate, *a* = 0.301 per day, and the deceleration rate of logistic tumour growth *b =* 1.01x10^-8^. We start the simulation procedure increasing the temporal step by one minute each time (Figure 7), and find that the tumour cell population actually become zero for extinction at 30.1 days (Figure 7A). One may notice that in this case the tumour cells are eliminated before the desired time duration, *t*_*p*_. This duration is different from the initially planned tumour elimination time *t*_*p*_, since, during simulation there may be time durations when a drug is stopped [this happens if its dose is calculated to have a negative or imaginary value [Figure 6, item (M)]], but the other drugs are increased with sufficient intensity to ensure tumour cell lysis at a substantial level. These increased therapeutic dosages can have nonlinear cell-killing effects and thus produce a shorter tumour elimination time than expected. It may be noted that we perform the computation for a time span of 20 times the extinction period (i.e. span of 800 days), a sufficiently long time to check permanency of regression. The regression appears to be lasting without any re-appearance of tumour cells, even though the therapy had been stopped much earlier (Figure 7A). It may be noted that for our plots or records, we take the computed cellular population values to have the next integer numeral, e.g. if *N, C, T* or T* is calculated out to be 1230.23 cells, the value is taken to be 1231 cells.

We also plot the blood concentrations of the cytotoxic T-cells, chemotherapeutic agent temozolomide and interleukin-2, required to eliminate the tumour (Figure 7B-7D). Also displayed are plots of the population of circulating lymphocytes and NK cells to check that there is no significant toxicity as side-effects of the therapy (Figure 7E-7F). We also observe that these values are also well within the corresponding bounds of the human host system (Table 2). Finally Figure 7G-7I show the injected dose rates, as required, day-wise, for each of the three therapeutic agents, that enables full tumour elimination. The circulating lymphocytes and NK cells takes around 500 days to reach the steady state values of 76 billion and 510,000 cells respectively. Note that these values closely corroborate with the stationary points solved theoretically earlier, which are 72 billion and 430,000 cells respectively [see eq. (6A) and subheading “Stationary condition of the system” there]. We find that the extreme values of these variables in the graphs are well within the bounds of the human host system (Table 2). For instance, the maximal dose-rate of temozolomide chemotherapy and of tumour-infiltrating lymphocytes are less than 10% and less than 1% respectively, of the upper bound of these agents in Table 2. Further, the cumulative dose of the interleukin (from the dose-rate graph calculated as area under the curve in Figure 7D), is below 1% of the interleukin upper bound in Table 2.

#### High-grade tumour

**Figure 8:**
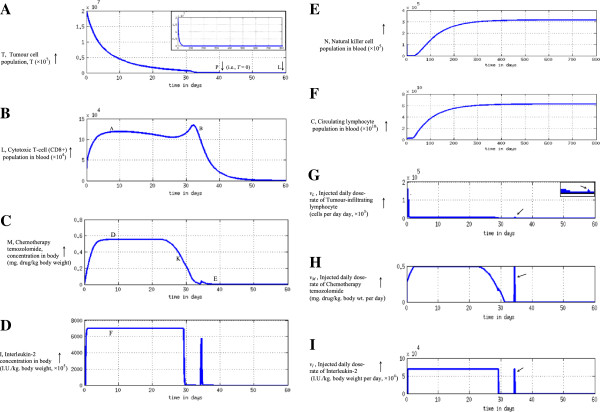
**Induction of complete elimination of low-grade tumour under multimodal therapy, inversely reconstructed with bias shift: astrocytoma III. (A) **Tumour cell population as therapy progresses, the population becomes zero at point P, after 40.11 days, the system is tracked for 800 days in the inset figure, to confirm the full extinction of tumour cells without any relapse over a period of 800 days. **(B)** The temporal variation of the Cytotoxic CD8+ lymphocyte population needed for full regression of tumour. One discerns the bimodal temporal dose-profile with two peaks at A and B. **(C)** The temporal profile of Temozolomide concentration necessary for tumour elimination. There is evidently an unimodal temporal dose-profile with the hump D, thence the chemotherapy level diminishes to point E; **(D)** The level of Interleukin-2 concentration required for extinction of the tumour cells. One notes the substantial level of interleukin (F), with truncation so as to have the level below the toxicity limit of interleukin in Table 2. **(E)** Natural killer cell population, *N* that does not cross the upper bound of physiological limits (Table 2). **(F)** Circulating lymphocyte population, *C* that can also be maintained below the upper bound of physiological limits (Table 2). **(G)** Injected daily dose-rate of Tumour-infiltrating Lymphocyte which is necessary for tumour elimination. An elevated pulse dosage injection essential at the latter part of the therapy, is delineated by arrow. **(H)** Injected daily dose-rate of Temozolomide chemotherapy that is requisite for the tumour regression. The pulse dosage injection required at the latter duration of the therapy, is shown by an arrow. **(I)** Injected daily dose-rate of Interleukin-2 that is needed for the extinction of tumour cells. An arrow shows the pulse dosage injection (point G) needed at the terminal portion of the therapy.

For the faster growing tumour such as astrocytoma or glioma grade III, we take tumour cell growth rate, *a =* 0.431, which is about 50% higher than the low-grade glial tumour discussed above. An initial malignant cell population (*T*_*0*_) of 2 × 10^7^, natural killer cell population of 10^5^, cytotoxic T-cell population of 5 × 10^4^ and circulating lymphocyte of 10^9^ cells were considered to simulate the system. The same tumour settling time and tumour elimination time-line is given, and a bias of T* = 1.8986 × 10^5^ cells is taken. The other parameters for the tumour system are given in Table 1. We proceed with the numerical simulation and observe that the tumour cells become extinct at 40.11 days (Figure 8A). Since the tumour cell proliferation rate, *a* value is higher here than the earlier tumour, the current tumour needs more treatment duration than the former. Likewise, we perform the computation for a time span of 800 days, to ensure the permanency of regression far across time. The values of the biological and pharmacological entities are displayed in Figure 8B-8I. The circulating lymphocytes and natural killer cells stabilize around 0.33 million and 62 billion cells respectively (Figure 8E-8F). These correspond to the stationary state populations predicted mathematically earlier. As in the earlier case of slow-growing tumour, one can also note the same for the rapidly-growing tumour:

(i) the dosages of the therapeutic agents drops to zero from the tumour extinction time onwards

(ii) none of the cell populations or dosing of therapeutic agents crosses the bounds (Table 2), and

(iii) the dosages of the three agents are approximately the same fraction of the upper bound (10%, 1% and 1%).

We also discern that the therapy period is considerably longer in high-grade tumour, basically since the tumour growth rate is about 50% more than the slow-growing tumour (value of parameter α, in Table 1). Further, since these therapeutic dosages are a much smaller fraction of the upper bounds, the dosage profile can be well tolerated by patients. Actually, the overall patterns of the graphs (Figures 7 and 8) are comparable, across both the high and low grade tumours.

### Robustness of tumour elimination procedure

The values of the parameters used are taken from Table 1. However these biological system parameters can vary across individuals, depending on the physiological and constitutional of the patient. Hence we need to check that the methodology is applicable even though the parameters may vary along a range. In order to test the versatility and robustness of our approach, we varied the initial immunological status (values of *N, C, L* cells) and tumour system parameters (values of tumour growth rate, α and carrying capacity, β). We performed 500 random simulations to check for complete tumour elimination, in both tumour grades. We found that tumour extinction for oligodendroglioma occurred in 100% cases if the variation was 0%, and in 98% of the cases as the coefficient of variation was increased to 10% (Table 4). For astrocytoma, the results are comparable. It has been known that the physiological parameters are generally kept constant homeostatically by organisms, with a 10% variation around the mean level [31]. Thus we can ascertain that the proposed approach may be able to induce tumour elimination in the large majority of cases.

**Table 4:** Robustness study with biological parameter variation

**Expt. No.**	**Coefficient of Variation**	**Percentage of success out of 500 cases**
1	0%	100%
2	1%	99.98%
3	2.5%	99.87%
4	5%	99.5%
5	7.5%	98.88%
6	10%	98.01%

### Unitary pattern in tumour regression process

If one compares the corresponding graphs in Figures 7 and 8, there are evident similarities in the temporal profiles of the therapeutic agents needed in both high-grade and low-grade tumours to enable elimination of malignancy. The common patterns valid across both tumours are elucidated below.

#### Profile A: terminal therapy pulse and cytotoxic lymphocyte persistence

One notes that (i) no injections of any of the three therapeutic agents are required any time after the time point of extinction of tumour, which does not recur later (Figure 7G-7I and Figure 8G-8I); (ii) the blood levels of the three therapeutic agents and the populations of the natural killer cells and circulating lymphocyte stay within the requisite limits, assuring that there would be no significant toxicity to the patient (Figure 7B-7F and Figure 8B-8F). One can make two pertinent observations from the simulation results. Firstly, for one to make the exponential decay curve of tumour cell population hit the *T* = 0 baseline (i.e. the *x*-axis of Figure 2B) at a definitive time point *t*_*p*_ , one needs to inject a terminal pulse of each of three therapeutic dosages before the end of treatment protocol in both the low-grade and high-grade tumours, as shown by arrows in the six graphs in Figure 7G-7I and Figure 8G-8I. The conjoint effect of the pulses of all three agents ensure that in the last stage, all the tumour cells in the exponential extremity of cell population decay curve, do become eliminated. Secondly, the persistence of the cytotoxic T lymphocytes in blood for a over a week after tumour has been eliminated (Figures 7B and 8B), can have a beneficial effect, such that it can act as a vigilant anti-tumour measure against recurrence, by acting for an appreciable time after tumour extinction. After that duration elapses, this lymphocyte population tapers off.

#### Profile B: common temporal paradigm of therapeutic agents needed

An insight into the mechanism of tumour regression may be obtained by investigating the commonalities in the pattern of the temporal variation in the blood levels of the therapeutic agents and population of the protective cells, which seem to be common across both high-grade and low-grade tumours. From Figure 7B-7D and Figure 8B-8D, we discern the following temporal patterns of the aforesaid entities, the pattern being similar for both rapidly and slowly growing tumours:

### (i) Temozolomide concentration (chemotherapy): monophasic activation pattern

**Figure 9:**
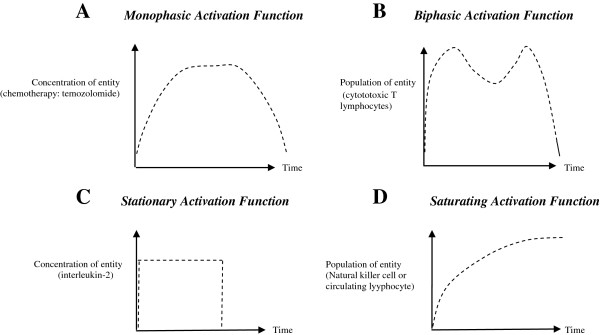
**The common pattern template of required activation functions of input factors associated with complete tumour regression. (A)** Unimodal intensity of chemotherapy (temozolomide). **(B)** Bimodal intensity of leucocyte immunotherapy (cytotoxic lymphocyte). **(C)** Uniform truncated intensity of cytokine immunotherapy (interleukin-2). **(D)** Saturating intensity of the tissue protective entities: natural killer cells and circulating lymphocytes.

The curve of *M* is unimodal for both low-grade and high-grade tumours, steadily rising initially, and later, gradually tapering off (Figures 7C and 8C). This chemotherapy concentration curve follows the usual pharmaco-kinetic curve of a customary drug administration: a unimodal hump, with gradually rising drug concentration to point D, and, later gradual concentration reduction (Figure 9A shows overall pattern).

### (ii) Cytotoxic T-lymphocyte (CTL) concentration (cytotherapy): biphasic activation pattern

This graph has bimodal peaks for both the high-grade and low-grade tumour (Figures 7B and 8B, arrows; Figure 9B). We observe that for tumour elimination, there is a need to have a peak of CTL, both, around the initial and the final phases of the therapy. The initial peak concentration in required to forcefully target and guide the trajectory of the cancer cell population curve (T) towards the zero baseline (Figures 7A and 8A). The final peak concentration of CTL, occurs before the time of tumour elimination, and is necessary to sufficiently eliminate the tumour cells and depress their population trajectory so that the same hits the zero baseline at the definitive selected time point P (Figure 1B). Depending on the tumour system dynamics, the height or activation-level of the second peak may be lower (Figure 7B), or higher (Figure 8B), than the height or activation-level of first peak. In case of Cytotoxic T-cell concentration, the first peak is due to therapeutic agents of tumour-infiltrating lymphocytes and interleukin, whose dosing starts as an impulse stimulus from the initial time. The impulse of these agents also enhance the generation of CTL population. The second peak in cytotoxic T-cells (point B in Figure 8B) occurs due to the later dip or decay of chemotherapy concentration along the hump of the *M* curve (point K in Figure 8C). The chemotherapeutic agent is toxic to and diminishes all the cellular constituents, including CTLs, and, hence, a decrease of chemotherapy induces a rise of CTL then. This same pattern of primary and secondary induction of T-cell population also occurs in the other tumour (Figure 7B-7C).

### (iii) Interleukin-2 concentration (immunotherapy): stationary activation pattern

This agent is needed at a substantially high level (at the upper bound, just below toxicity level), so as to induce a higher level of immune activation that would enable complete elimination of glioma cells (Figures 7D, 8D and 9C). Indeed it is well known from clinical experience in neurooncological immunotherapy that interleukin-2 administered at significantly augmented dose, induces long-lasting immunomodulation to act against those malignant cells that bypass usual therapeutic intervention [36]. Actually, the graph for the high- and low-grade tumours has rapidly rising high amplitude, as the interleukin-2 level is truncated and kept stationary within toxicity limit.

### (iv) Circulating lymphocyte population: saturating activation pattern

This population initially increases and then plateaus in both tumours (Figures 7F, 8F and 9D), to approach the saturation level of the long-term steady state as mentioned earlier.

### (v) Natural killer cell population: saturating activation pattern

Similar to the Circulating lymphocytes, the NK cells show saturation behaviour for both high and low grade tumours (Figures 7E, 8E and 9D).

#### Experimental, biological and clinical corroboration

To justify, the *modus operandi* of our approach is based on the following aspects: firstly, the tumour elimination process is a manifestation of exponential diminution with bias shifting; secondly, the procedure works on fundamentally two complimentary modes of tumour cell reduction:

(a) Reducing the tumour cell proliferation, as by chemotherapy or chemical alkylation (DNA damage);

(b) Increasing the tumour cell lysis by anti-tumour lymphocytes, which are activated by cytokine modulation (for instance tumour infiltrating lymphocytes, interleukin-2 etc.).

Further, the model developed shows that to induce tumour regression, the three antitumour entities should have three distinct temporal profiles: (1) biphasic intensity for lymphocyte activation, (2) monophasic intensity for activation of chemomodulative DNA damage (chemical alkylation), (3) stationary intensity of cytokine activation (interleukin-2). The experimental studies in animals [37-39] and clinical situation [40,41] corroborate these findings. Thus our results show that complete elimination of tumour can be attained by (i) *the five-pattern profile*: activation of antitumour lymphocyte (bimodality), chemomodulative DNA damage (unimodality), interleukin (stationary), natural killer cell (saturation), and circulating lymphocyte (saturation), (ii) *the two kinetic conditions*: bias shift and exponential decrementing dynamics [the tumour trajectory formula of eq. (4)].

### Translational applicability

There are two aspects where the approach could be improved. Firstly, one can increase the system robustness, which diminishes as the individual patient-specific fluctuations increases (see earlier subsection on Robustness). For real-time implementation, we can use the neuro-adaptive controller, which can reliably follow the desired mathematical trajectory of the tumour cell population curve (Figure 2B), and can be well adapted to different values and fluctuations of biological parameters of different patients. We have used such a controller in devising imatinib chemotherapy dosing in chronic myeloid leukaemia [16], and 100% robustness was obtained even when the maximal tumour density varied from 150,000 to 400,000 cells/mm^3^, indicating about 250% variation on the baseline level. Secondly, for proper monitoring of a patient, the declining tumour load *T* can be weekly or semiweekly estimated non-invasively, by MRI amide-proton transfer imaging, which maps the cell proliferation intensity by amide mobility [42]. This emerging technology holds high potential, as this method is an efficient one to distinguish between various oncological conditions such as tumour recurrence, tumour haemorrhage or tumour necrosis.

**Figure 10:**
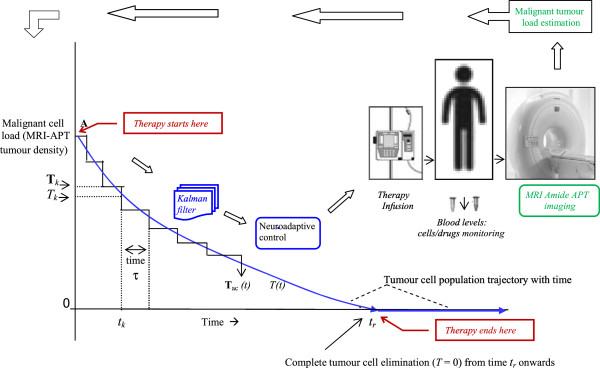
**Clinical implementation of tumour elimination procedure, using MRI-based tumour-load monitoring method at weekly time intervals.** The multimodal therapy starts from point A and its effect on the malignant tissue is monitored by estimating the tumour load (correlate of tumour variable *T*) using MRI amide proton-transfer imaging, done at time intervals of τ. The blood levels of the therapeutic agents and of its cell populations (circulating lymphocytes, natural killer cells, cytotoxic T-cells etc.) are also measured then. These data are used to give corrective feedback to the therapy system in case there is incongruity between the actual trajectory and the mathematically calculated trajectory desired. Data sampling at τ and any uncertainty or instability of the biological parameters, are taken care by Kalman’s filtering and neuroadaptive control which actuates the infusion pump of the therapeutic inputs to the patient.

In Figure 7A the tumour cell diminution trajectory is shown for the subject in question, the cell population being mathematically derived given the physiological parameters. It would be preferable if one could check this mathematically obtained curve *T(t)* with the actual real-life tumour cell population **T***(t)* as the treatment process goes over months, the actual population **T***(t)* being estimated radiologically at suitable discrete time intervals of τ (Figure 10). The aim is to have correction-making feedback so that the actual realistic curve **T** tracks and follows the desired computationally-optimized curve *T.* In case if there is a discrepancy at a particular time point *t*_*k*_, between the actually-measured value and the mathematically-predicted value (i.e. between **T** and *T* at time point *t*_*k*_), then the value at that moment of the actual number of tumour cells **T**_*k*_ can be used as the input value of tumour cell population at time point *t*_*k*_ in the simulation procedure for going to the next time point *t*_*k+1*_ as per the procedure in Figure 6. Thereby, the new dosages of the drugs can be calculated for reaching the same target, namely zero population of tumour cells. The result is that the actual trajectory of the diminution of the tumour cell population becomes a discrete approximation of the continuous mathematical curve, with the time interval τ (Figure 10). Nevertheless, the final target of zero tumour cell population is attained.

For monitoring while the therapy is in progress, one need to have at suitable time intervals, the overall measurements of the serum temozolomide and interleukin-2 levels, as well as the populations of cytotoxic T-cells, natural killer cells and circulating lymphocyte in the blood (Figure 10). Only approximate values of these parameters or of the tumour cell population are required, as even large variations of these parameters can be accommodated by the neuro-adaptive controller mentioned above. During practical implementation, it may not be possible to measure all the biological parameters, neither a parameter can be estimated at all the desired time points as there may be discontinuous or missed sessions. Here, one can use suitable quantitative procedures as the Kalman’s technique or constraint filter method which has been used in immunology to estimate the likely values of biological parameters at the discrete or missed sessions, given their measured values in other sessions [43,44]. Thus, it would suffice to monitor the tumour parameters weekly or twice-weekly, to enable the therapy control system tracking and adapting function, as demarcated in Figure 10.

One may observe that during therapy (Figures 7E-7F and 8E-8F), the circulating lymphocytes and natural killer cells increase but there is no hazard, as the populations are within the upper physiological limits of Table 2. Moreover, for making the methodology more suited to personalized medicine so that we can pre-select the most sensitive drugs beforehand, we can utilize the emerging method of tumour-graft technology whereby one can grow a therapeutically faithful model of the individual patient’s tumour biopsy tissue on experimental mice [45]. Tumour graft platforms can test different drug combinations, and pre-select the most sensitive drug before starting the treatment, the accuracy of selecting effective drug molecule being 86%. Lymphocytic (CTL) immunomodulation can be of tactical utility, as it exhibits a range of unique behaviors [46], that chemotherapeutic drugs cannot, such as (i) the cells can migrate to the antigen-bearing primary or secondary growths of tumour, even in hidden tissue depths, (ii) CTLs can continue to multiply automatically in response to immunogenic proteins of malignant cells, until all those cells become extinct.

## Conclusion

We have formulated the distinctive dose-time relationship of chemotherapy and of immunotherapy (interleukin-2 and cytotoxic lymphocyte), such that their orchestrated functioning, that incorporates a biphasic temporal profile for T-cell, ensures that all the tumour cells are eliminated within a desired time. Excess toxicity to the host is avoided, as the circulating lymphocyte and natural killer cells in blood are protected. The approach is patient-specific as the formulation depends on the tumour load, the levels of cytotoxic T lymphocytes, cytokine interleukin, natural killer cells and circulating lymphocytes. All these parameters vary with the individual patients, and hence the different therapeutic dose-time profile obtained for each specific patient will be optimally suited from her. The formulation put forward use of an innovative approach of bias shift, control systems analysis, performance cost minimization and inverse construction of drug input. The limitation of the proposed model is that we have not considered the effect of tumour cells resistant to the chemotherapy drug and also not taken care of angiogenesis process where the drug permeability to the tumour cells will hinder. A broad-based gamut of findings from animal experimentation and clinical investigations are shown to corroborate with the tumour extinction approach developed. An unanticipated but noteworthy finding is the importance of giving a terminal pulse of the therapeutic agents before the end of the therapy, so that all the tumour cells become extinct and there is no extra drug-induced toxicity, the natural killer cells and circulating lymphocytes being within the physiological limits. This is in contrast to the generally prevailing view in clinical medicine, which advocates tapering off of the therapy in the later stages. This tapering may cause tumour recurrence in clinical praxis, as there is no intensive spike of the therapeutic agents to eradicate all the malignant cells.

To summarize, information from temporal dynamics of both the endogenous and exogenous tumour regression has been used to explore the mechanism and elucidation of integrative functioning of various therapeutic modalities, whose combined effect eliminates the malignant tumour as corroborated with experimental findings. The method proposed can be of wide-ranging application, and can be adapted for application to conditions where there is involvement of chemotherapy and/or immunotherapy. The procedure delineated is also applicable to other tumour systems, as it offers a principled approach to tumour containment and thus an incisive prospect for probing towards further biological and clinical situations.

## Endnotes

^***a***^ ***Clarifications on upper/lower limits of biological and therapeutic parameters (Table*** 2 ***).***

*Circulating lymphocyte population:* The normal blood volume under active circulation is 3.5-4.5 litres. The higher limit of lymphocytes in individuals can be up to 100 × 10^9^ cells/litre [26]. Taking the upper bound of the blood volume, this translates to the higher bound of lymphocyte in a person to be 4.5 × 10^11^ cells. In contrast, the lowest lymphocyte count for patients for duration of 6–12 months across therapy can be 663–1160 cells/μl [27]. Taking the lesser value of the cell count and the lower level of blood volume, we get minimal bound of lymphocytes as 2.32 × 10^9^ cells, which the patient can tolerate up to 6 months.

*Natural Killer Cells population:* The upper limit of NK cell is 13% of lymphocyte population, with CD56/CD16 surface protein being the marker for these lymphocytes [28]. As the higher bound of lymphocyte population in the earlier paragraph is 4.5 × 10^11^ cells, we have the maximum value of NK cell population in the individual to be 5.85 × 10^10^ cells. On the other hand, the lower limit of NK cells (CD56/CD16 lymphocytes) is 0, occurring in people having natural killer cell deficiency condition [29], and a period of 3½ months have been noted for elapse of this condition, before considerable infection can set in [29]. Hence we also mention this time duration for the NK cells lower bound.

*Tumour-specific Cytotoxic (CD8+) T-cell population:* The upper limit of this cell population for a patient is 20150 cells/mm^3^[25]. Using a blood volume of 4.5 litres, the total T-cell (CD8) population will be 6.05 × 10^10^ cells. Furthermore, tumour-specific cytotoxic T-cells, that are specifically active against a particular malignant lesion, has been known to come into play if the tumour is present, and to decay away if the tumour undergoes elimination [30]. Hence, one mentions the lower bound of these T cells to be 0.

*Temozolomide chemotherapy infusion dosage rate:* Maximum daily dosage [1] of temozolomide is 200 mg/m^2^/day, i.e., 4.45 mg/kg body. wt. per day. One may choose not to give it, so the lower limit is 0.

*Interleukin-2 immunotherapy infusion dosage rate:* Maximum infusion given is 7.2 × 10^4^ International Units (I.U.)/kg/day [1]. The lower bound can be set to nil, as above.

*Tumour-infiltrating lymphocytes (TIL) immunotherapy cumulative dosage:* The maximum cumulative dose for a patient over the whole duration of therapy is 13.7 × 10^10^ cells [25]. Likewise, the lower limit is zero.

^***b***^ ***Determination of initial number of the cells for numerical experimentation.***

*Tumour cells:* The number of cells in a tumour which becomes clinically detectable is 10^8^ occupying a volume of 1 cm^3^, out of these 10^7^ cells are malignant, while the rest are stromal cells [47]. For our quantitative experimentation, we take double the amount of tumour cells to have a safety factor of 2, thus giving 2 × 10^8^ malignant cells as our initial condition.

*Circulating lymphocytes:* We also note the range of normal values: leucocyte count = 4000 to 11000/mm^3^, the fraction of lymphocytes are 15-40%, and actively pulsating blood volume under circulation is 3.5-4.5 litres. To be cautious for ensuring tumour regression, we will consider the lower values in the range. By multiplying the requisite aforesaid values, we have the circulating lymphocyte population in the person as 2.1 × 10^9^ cells. Again to be on safer side, we take half of the population for our study (i.e. 1.05 × 10^9^ cells, say 10^9^ cells).

*Natural killer cells:* The fraction of NK cells is 1-13% of circulating lymphocytes [28], we take the lower value for calculation, and use the circulating lymphocyte value of 1 × 10^9^ cells given above. Further, there is available data on the antitumour effector factor of NK cells during study of endogenous tumour regression, namely 1:50 as target tumour cell: effector NK cell ratio, i.e. a value of 2% [48]. Multiplying these values, we get the effective population of tumour-targeted NK cells as 2 × 10^5^ cells. As earlier, we will consider half this value to be on the safe side, i.e. NK cell population = 10^5^ cells.

*Cytotoxic T-cells:* The fraction of cytotoxic CD8+ T-cells is 1-3% of circulating lymphocytes [49]. The cytotoxicity factor of activated tumour-infiltrating cytotoxic T cells (CTL) is 9.5% as regression process is underway [48]. One also knows that if the tumour is invasive and spreads, the fatigue factor come into force, which can cause the CTL efficiency (as estimated by cytokine production) to decrease to 12.2% of the level as compared to when there is no invasion of tumour [50]. Using these factors, we arrive at the effective population of cytotoxic T cells as 1.15 × 10^5^ cells, say 10^5^ cells. As per the lower end, we have taken half, i.e. 5 × 10^4^ cells as the CTL population.

## Competing interests

The authors declare that they do not have any competing interests.

## Authors’ contributions

The research was conceived, designed and planned by PR and RP. The modelling and numerical experiments were performed and the biological studies investigated by SK, VPSR, CK, RP and PR. All authors examined and evaluated the data. SK, VPSR and PR wrote the manuscript, with participation from CS and RP. All authors read and approved the final manuscript.

## Supplementary Material

Additional file 1 Supporting Methods. Click here for file

Additional file 2 Supporting Analysis. Click here for file
