# Supplementary material for: A patient-specific therapeutic approach for tumour cell population extinction and drug toxicity reduction using control systems-based dose-profile design
Source: Theor Biol Med Model. 2013 Dec 26;10:68. doi: 10.1186/1742-4682-10-68 (PMC3896771; doi:10.1186/1742-4682-10-68)
Supplement: Additional file 1 — Supporting Methods. [file 1742-4682-10-68-S1.pdf]

ADDITIONAL FILE 1

SUPPORTING METHODS

A.1	Quantitative Formulation of Tumour Dynamics	Page 1
	References for Supporting Methods	Page 3

A.1 Quantitative Formulation of Tumour Dynamics

Denoting the temporal derivatives by primed symbols, the rate of change of tumour cell population is:

***Tumour cell dynamics**:* $T' = \frac{dT}{dt} = \underbrace{aT(1-bT)}_{\text{Logistic tumour growth}} - \underbrace{cNT - DT}_{\text{Tumour lysis by NK cell \& T cell}} - \underbrace{k_T(1-e^{-M})T}_{\text{Tumour lysis by chemotherapy (saturation behaviour)}}$ (A1)

Here the tumour growth is logistic, as per the term $aT(1-bT)$ with a as growth rate and b as decelerating rate, while the term NT represents the interaction between tumour and NK cell populations, the term DT represents the tumour lysis by cytotoxic T-cells (D is a parameter whose formula will be given later), and the last term above $(1-e^{-M})$ is taken to be saturation term that accounts for the chemotherapy fractional cell kill. Note that at relatively low concentrations of drug, the kill rate is linear, while at higher drug concentrations, the kill rate plateaus. It may be underscored that the equations being deliberated here recollect the experimentally-based immunological reaction-rate framework demarcated by recent analyses [A1-A3], which has also been empirically validated [A4] (the references are at the end of this file). The approach also marshals the findings of animal studies and human clinical trials [A5-A7], and harnesses the models utilized in cellular population biology, that encompasses reaction kinetics and saturation processes as logistic functions and Michaelis-Menten type and Hill-type of interactions.

The rate of change of natural killer (NK) cell population is furnished by

Natural killer cell dynamics: $N' = \frac{dN}{dt} = \underbrace{eC}_{\text{NK cell birth from circulating cells}} - \underbrace{fN}_{\text{NK cell death by senescence}} + \underbrace{g\frac{T^2}{h+T^2}N}_{\text{NK cell recruitment by tumour cells}} - \underbrace{pNT}_{\text{NK cell de-activation by tumour cell debris}} - \underbrace{k_N(1-e^{-M})N}_{\text{NK cell lysis by chemotherapy}}$ (A2)

In the above equation*, the growth of NK cells is associated with the overall immune health gauged by the population of circulating lymphocytes (term eC). This enables for the suppression of stem cells during chemotherapy, which in turn diminishes the circulating lymphocyte population, which in turn, affects the generation of NK cells (resultant term $-fN$), hence the gross NK cell growth term is $eC - fN$. The expression $NT^2/h+T^2$ is a modified Michaelis-Menten term, providing a saturation effect in tumour models to govern cell-cell interactions. The term pNT is the inactivation of the cytolytic potential occurring when an NK cell has interacted with tumour cells, while the last term $(1-e^{-M})$ is taken to be saturation term to represent the chemotherapy fractional NK cell kill, the factor k_N being a proportionality constant.

* Equations of the Additional Files annexure are prefixed by alphabets, such as eq.(A1), while equations of the initial textual matter are referred without a prefixed letter, namely as eq.(1).

The rate of change of $CD8^+$ T cells can be arrived as

Cytotoxic T-lymplocyte (CTL) dynamics: $CD8^+$ T-cells:

$$L' = \frac{dL}{dt} = -mL + j\frac{D^2T^2}{k+D^2T^2}L - qLT + (r_1N + r_2C)T - uNL^2 - k_L(1-e^{-M})L + \frac{p_I\,LI}{g_I+I} + v_L(t) \quad \text{(A3)}$$

T-cell death (senescence) | T-cell recruitment by tumour cell | T-cell de-activation by tumour cell debris | T-cell activation by NK & circulating cells | T-cell suppression: NK cell dependent | T-cell lysis: chemotherapy | T-cell stimulation by Interleukin-2 | Injected TIL: T-cell therapy

Regarding eq.(A3), the cell decay rate for these cells is represented by the natural death rate (negative term *–mL*), and none of these cells are present in the absence of tumour cells. The term $D^2T^2/k+D^2T^2$ represents the CTL cell recruitment by the lysed tumour population originating from the Tumour cell-CTL cell interaction term *D(T, L)* that also appears in eq.(A1). The term *qLT* is the inactivation of cytolytic ability, occurring when CTL cell has interacted several times with tumour cells. The term r_1NT represents that CTL cells that are recruited by the debris of tumour lysis induced by NK cells, while the r_2CT term is taken since the immune system is also stimulated in the presence of tumour cells to produce more CTL cells. The recognition of these tumour cells by the CTL cells is proportional to the average number of encounters between circulating lymphocytes and the tumour.

To delineate the formulation in eq.(A3) further, we note that the inactivation term uNL^2 represents the regulation and suppression of CTL cell activity, which occurs when there are very high levels of activated CTL cells without responsiveness to cytokines present in the system. The term $(1\text{-}e^{-M})$ is taken to be the saturation term that represents the chemotherapeutic fractional kill of the CTL cells, and k_L is a proportionality constant. The term (p_ILI/g_I+I) represents the activation of CTL cells by Interleukin-2 immunotherapy, which is in Michaelis-Menten form, while the last term $v_L(t)$ represents the injected dose rate of the immune cell therapy component (cytotherapy as drug agent) consisting of the antigen-specific Tumour infiltrating lymphocyte (TIL), that activates the CTL cells to lyze the tumour cells.

The rate of change of circulating lymphocytes can be inferred as

Circulating lymphocyte dynamics:

$$C' = \frac{dC}{dt} = \alpha - \beta C - k_C(1-e^{-M})C \quad \text{(A4)}$$

Birth of circulating cells | Death of circulating cells (senescence) | Lysis of circulating cells by chemotherapy

Observe that the circulating lymphocytes are generated at a constant rate (α) from bone marrow, and that each cell has a natural lifespan (death rate β). The term $(1\text{-}e^{-M})$ is the saturation term that represents the chemotherapeutic fractional kill of circulating lymphocyte cells, with k_C being a proportionality factor.

The rate of change of chemotherapy drug concentration (temozolomide) is given by

Chemotherapy dynamics:

$$M' = \frac{dM}{dt} = -\gamma M + v_M(t) \quad \text{(A5)}$$

Chemotherapy washout | Injected chemotherapy

Since the chemotherapy drug after intervention will be eliminated from the body over time at a rate proportional to its concentration, the decay term is γM. Further, the term $v_M(t)$ represents the injected chemotherapy dosage.

The rate of change of concentration of immunotherapy agent (interleukin-2) is provided by

Immunotherapy dynamics: $I' = \frac{dI}{dt} = -\mu_I I + v_I(t)$ (A6)

↓ Interleukin elimination ↓ Injected interleukin

The immunotherapeutic drug agent (interleukin-2) after injection will be eliminated from the body over time at a rate μ_I proportional to its concentration I, while the term $v_I(t)$ represents the injected dosage rate of the immunotherapy.

The interaction between the CTL cell population and tumour cell population [e.g. D in eq.(A1)] is considered to be Michaelis-Menten form. Using d as a constant of proportionality, we can represent D as:

Tumour cell – Cytotoxic T cell interaction: *D(T,L):* $D = d\frac{(L/T)^l}{s+(L/T)^l}$ (A7)

↓ Saturation function (T cell – Tumour cell transaction)

References for Supporting Methods

A1. de Pillis L, Gu W, Radunskaya A: **Mixed immunotherapy and chemotherapy of tumour: Modeling, application and biological interpretations**. *J Theor Biol* 2006, **238:** 841–862.

A2. Kuznetsov V, Makalkin I, Taylor M, Perelson A: **Nonlinear dynamics of immunogenic tumours: parameter estimation and global bifurcation analysis.** *Bull Math Biol* 1994, **56:** 295-321.

A3. Kirschner D, Panetta J: **Modeling immunotherapy of the tumour–immune interaction**. *J Math Biol* 1998, **37:** 235–252.

A4. de Pillis L, Radunskaya A, Wiseman C: **A Validated Mathematical Model of Cell-Mediated Immune Response to Tumour Growth.** *Cancer Res* 2005, **65(17):** 7950-58.

A5.Dudley M, Wunderlich J, Robbins P *et al*: **Cancer regression and autoimmunity in patients after clonal repopulation with antitumour lymphocytes.** *Science* 2002, **298:** 850-854.

A6. Diefenbach A., Jensen E, Jamieson A, Raulet D: **The Rael and H60 ligands of NKG2D receptor simulate tumour immunity**. *Nature* 2001, **413:** 165-171.

A7. Lanzavecchia A, Sallusto F: **Dynamics of T-lymphocyte responses: intermediates, effectors, and memory cells.** *Science* 2000, **290:** 92–97.
