# Supplementary material for: A patient-specific therapeutic approach for tumour cell population extinction and drug toxicity reduction using control systems-based dose-profile design
Source: Theor Biol Med Model. 2013 Dec 26;10:68. doi: 10.1186/1742-4682-10-68 (PMC3896771; doi:10.1186/1742-4682-10-68)
Supplement: Additional file 2 — Supporting Analysis. [file 1742-4682-10-68-S2.pdf]

ADDITIONAL FILE 2

SUPPORTING ANALYSIS

B.1 Adjustment of therapeutic weights Page 1

Case I: Chemotherapy Page 1

Case II: Immunotherapy - Tumour Infiltrating Lymphocytes Page 3

Case III: Cytokine – Interleukin 2 Page 6

B.2 Computation of therapeutic weights Page 8

B.3 Summary of procedure to obtain the therapeutic weights Page 10

Table I Formulation of Boundary Parameters that provide Therapeutic weights Page 11

B.4 Sub modal Protocols Page 12

Two drug formulation Page 12

One drug formulation Page 16

References for Supporting Analysis Page 17

B.1 Adjustment of therapeutic weights

The adaptable parameters are the therapeutic weights of the antitumour agents, acting at the two stages:

(i) Tumour Cell compartment: The therapeutic weights are $r_{T1}, r_{T2}, r_{L1}, r_{L2}$, which respectively denote the antitumour efficiency weight of temozolomide and cytotoxic T-cells respectively.

(ii) Cytotoxic T-cell compartment: the therapeutic weights are r_{L_1}, r_{L_2}, which respectively denote the T-cell activation weight of interleukin-2 and tumour-infiltrating lymphocytes respectively.

Since the tuning parameters deal with actual cellular and biochemical processes and flow rates, the tuning parameter values cannot have imaginary values and must be greater than or equal to zero. The value of the tuning parameters $r_{T1}, r_{T2}, r_{L1}, r_{L2}$ which appear in derivation are not chosen to be fixed; instead they are chosen by the mathematical conditions elucidated below. They are continually adjusted as the therapy progresses, so as to minimize the drug intensity and toxicity to the patient, and yet at the same time enforce the tumour cell population to become zero. For such optimization problems, it transpires that significant characteristic to be attended to is the ratio $r_{T1} : r_{T2}$ [B1] (References are given at the end of this file). Thus, we can take the parameter r_{T1} to have a normalized value of unity (i.e. $r_{T1} = 1$), thereby the task is to suitably choose or optimize the value of the other tuning parameter r_{T2}. Likewise, the same logic applied to the ratio of $r_{L1} : r_{L2}$. Thus, we can have the values of two of the parameters as:

$$r_{T_1} = 1 \quad \text{and} \quad r_{L_1} = 1. \qquad \text{eq.(B1)}$$

We elaborate below the bounds of the other two tuning parameters (r_{T_2}, r_{L_2}) and how these are related to:

(i) the pharmacological efficiency factors of the three drugs (U_M, U_L, U_I) [intermediate control variables];

(ii) the injected dose-rates of the drugs, $v_M(t), v_L(t), v_I(t)$ [final control variables].

Case I: Condition for the using Chemotherapy Temozolomide injection [v_M]:

We consider temozolomide which acts through the tumour cell population compartment (Figure 1) where the toxicity cost J_T is related to the tuning parameters or weights, r_{T1} and r_{T2} [see the subsection after eq.(15) of the text]. We now attempt to find the values of the tuning parameter range for effective functioning of the chemotherapy, this span of values will give room for altering the tuning weight to enable complete tumour elimination.

Limit conditions of the temozolomide clinical efficiency factor U_M

Upper limit of U_M:

From eq.(A14) † the desirable blood level of temozolomide is

$$M^* = -ln(1 - U_M). \qquad \text{(B2)}$$

Since M^* needs to avoid having imaginary values, then temozolomide's clinical efficiency parameter, $U_M < 1$, since the logarithm is not defined for zero or negative values. Thus, the upper limit of U_M is 1 .

† Any equation number prefixed by 'B' [such as eq.(B14)] refers to Additional File 2: Supporting Methods.

Lower limit of U_M:

Now, we recall eq. (A30), $v_M(t) = \gamma M - k_M(M - M^*)$. Since $v_M(t)$ is the drug injection rate, it cannot be negative and hence $v_M(t) \geq 0$. Substituting this value in the equation of the earlier line,

$$\gamma M - k_M(M - M^*) \geq 0 \qquad \text{(B3)}$$

Using M^*value from aforesaid eq. (B2) in eq. (B3),

$$\gamma M - k_M(M + ln(1 - U_M)) \geq 0$$

Transposing the above, we have the lower limit of U_M, viz.

$$U_M \geq 1 - exp[(\gamma M/k_M) - M] \qquad \text{(B4)}$$

Limit conditions of the therapeutic weight factor r_{T_2} :

Lower limit of r_{T_2}:

Earlier, during the toxicity cost J_T minimization [eq.(A10)], we noted a condition relating U_M with the tuning parameters r_{T_1} and r_{T_2}, namely

$$U_M = b_T\, g_{T_1} / r_{T_1} G, \qquad \text{(B5)}$$

where G = [$(g_{T_1}{}^2 / r_{T_1}) + (g_{T_2}{}^2 / r_{T_2})$]. Substituting this expression of into eq.(B5), we have:

$$b_T\, g_{T_1} / r_{T_1} G \geq 1 - exp[(\gamma M/k_M) - M] \qquad \text{(B6)}$$

Recollecting that the relaxation decay parameter b_T has the form of a negative term from eq.(13) of the text, and by transposing eq.(B6), we obtain the lower limit of r_{T_2}:

$$r_{T_2} \geq g_{T_1}{}^2\; r_{T_1} / [\{ b_T\, g_{T_1} / 1 - exp\,(M\gamma/k_M - M)\} - g_{T_1}{}^2] \qquad \text{(B7)}$$

where, apropos eq.(13) in the text, we know that $b_T = - k_T(T - T^*) - f_T(X_n) = [\, aT(1 - bT) - cNT\,]$.

Upper limit of r_{T_2}:

We recollect from the last two subsections that $U_M < 1$ and $b_T \leq 0$, and using eq.(B6), we have

$$b_T\, g_{T_1} / r_{T_1} G < 1 \qquad \text{(B8)}$$

where G, as before, stands for [$(g_{T_1}{}^2 / r_{T_1}) + (g_{T_2}{}^2 / r_{T_2})$]. Transposing eq.(B8), we obtain:

$$r_{T_2} < g_{T_2}{}^2\; r_{T_1} / (b_T\, g_{T_1} - g_{T_1}{}^2). \qquad \text{(B9)}$$

From eq. (B7) and (B9), we arrive at the range of values that r_{T_2} can take:

$$g_{T_1}^{\ 2}\ r_{T_1} /\ [\{\ b_T\, g_{T_1} /\ \ 1 - exp(M\gamma/k_M\ -\ M)\} - g_{T_1}^{\ 2}\ ]\ \ \leq\ r_{T_2}\ <\ g_{T_2}^{\ 2}\ r_{T_1} / (b_T\, g_{T_1} -\ g_{T_1}^{\ 2}). \quad \text{(B10)}$$

Let A and B be the left hand side and right hand sides of the inequality (B10) ; i.e.

$$A = g_{T_1}^{\ 2}\ r_{T_1} /\ [\{\ b_T\, g_{T_1} /1 - exp(M\gamma/k_M - M)\} - g_{T_1}^{\ 2}\ ] \quad \text{(B10-A)}$$

$$B = g_{T_2}^{\ 2}\ r_{T_1} / (b_T\, g_{T_1} -\ g_{T_1}^{\ 2}). \quad \text{(B10-B)}$$

We observe that actually the therapy-initiated tumour effect term b_T can be either be negative implying tumour cell population decrease ($b_T \leq 0$), or be positive, indicating tumour cell population increase ($b_T \geq 0$). Therefore, if the situation of tumour cell regression occurs, i.e. for $b_T \leq 0$, we have the range of r_{T_2} as:

$$A \leq r_{T2} < B \quad \text{(B11)}$$

On the other hand, if the complimentary situation of tumour cell progression occurs, i.e. $b_T \geq 0$, then

$$A \geq r_{T2} > B \quad \text{(B12)}$$

The toxicity weight of the cytotoxic T-cell (r_{T_2}) should have values within the above range, and this requirement is a sufficient condition for tumour regression when considering the use of chemotherapy temozolomide.

Case II: Condition for the using Tumour infiltrating lymphocyte injection [v_L]:

We here attend to Tumour-infiltrating leucoyte, that actuates the cytotoxic T-cell level that acts via the tumour cell population compartment (Figure 1) where the toxicity cost J_T is related to the tuning parameters or weights, r_{T1} and $r_{T2} r_{T2}$ [refer to the subsection after eq.(15) of the text]. We now attempt to find the values of the tuning parameter range for effective functioning of the chemotherapy, this span of values will give room for altering the tuning weight to enable complete tumour elimination.

Limit conditions of the cytotoxic T-cell efficiency factor U_L

Upper limit of U_L:

As per eq.(A14), the desirable cytotoxic T-cell population level is

$$L^* \ = \ \sqrt[l]{[\ s\, T^l\, U_L / (d - U_L)\ ]}. \quad \text{(B13)}$$

Since L* is efficient in facilitating tumour elimination, its clinical efficiency U_L cannot be negative, i.e. $U_L \geq 0$ in the numerator in eq.(B13). Further, as L* is a positive finite cellular population, the

denominator cannot be zero nor negative, which respectively imply that $U_L \neq d$ and $d - U_L > 0$ (i.e., $U_L < d$). Note that the upper limit of U_L, namely the parameter d, is the saturation level of fractional tumour cell kill by cytotoxic T-cells (eq.A7), thus d is positive.

Lower limit of U_L:

Considering the above paragraph , we can write the range of U_L as:

$$0 \leq U_L < d \qquad \text{(B14)}$$

Thus the lower limit of U_L is 0. Now, to attend to the rage of r_{T_2}, we consider eq.(A11), whereby we get $U_L = b_T\, g_{T_2} / r_{T_2}$ G, where G = [$(g_{T_1}{}^2 / r_{T_1}) + (g_{T_2}{}^2 / r_{T_2})$]. Using this U_L in eq. (B14):

$0 \leq b_T\, g_{T_2} / r_{T_2}\, G < d.$

Thus, $b_T\, g_{T_2} / r_{T_2}\, G > 0$, and $b_T\, g_{T_2} / r_{T_2}\, G < d$. Taking reciprocal, these two inequalities imply:

$$r_{T_2} \leq \infty \qquad \text{and} \qquad b_T\, g_{T_2} / d\, G < r_{T_2}$$

This means $$b_T\, g_{T_2} / dG < r_{T_2} \leq \infty \qquad \text{(B15)}$$

Bounds of antitumour effect term of therapy (b_T):

We now attend to the two bounds in eq.(B15).

Put C = $b_T\, g_{T_2}/dG$, and let D = ∞ (B15-A)

So, from eq.(B15), the following emerges:

for tumour regression case, i.e., $b_T \leq 0$, we have $C < r_{T2} \leq D$ (B16)
while for tumour progression case, i.e., $b_T \geq 0$, one gets $C > r_{T2} \geq D$ (B17)

Thus, eq. (B16) and (B17) are the conditions for the desired population of cytotoxic T-cell L^* to have a real number value.

Bounds of T-cell activation effect term of therapy (b_L):

The symbol b_T signifies the total cytotoxic T-cell activation effect by the two immunotherapeutic inputs: the interleukin efficiency term U_I and the tumour-infiltrating lymphocyte administration term v_L .

Since the therapy dose rates $v_L(t)$ and $v_I(t)$ depends on this desired cytotoxic T-cell value L^* (Figure 1), one can posit that eq. (B16) and (B17) are the necessary condition for administering the tumour-infiltration leurocyte and interleukin dose-rates, $v_L(t)$ and $v_I(t)$ respectively.

Further, we have $$v_L(t) \geq 0 \qquad \text{(B18)}$$

because the dose-rate $v_L(t)$ cannot be negative. From eq.(42), we recapitulate the expression

$$v_L = b_L / r_{L_2} \mathrm{H}, \qquad \text{(B19)}$$

where H = [(1/ r_{L_1}) + (1 / r_{L_2})], H being a positive quantity as the therapeutic weighting factors, r_{L_1} , r_{L_2} are positive. Substituting eq.(B19) in eq.(B18), we have

$$b_L / r_{L_2} \mathrm{H} \quad \geq \quad 0 \qquad \text{(B20)}$$

Since r_{L_2} and H are both positive, eq.(B20) implies that we should need that

$$b_L \geq 0 \qquad \text{(B21)}$$

Recollecting the tracking dynamics aspect, i.e. eq.(A23) and (A21):

$$b_L = -f_L(X) - k_L(L - L^*). \qquad \text{(B22)}$$

where $f_L(X) = -mL + j\frac{D^2T^2}{k+D^2T^2}L - qLT + (r_1N + r_2C)T - uNL^2 - K_L(1 - e^{-M})L$ (B23)

Substituting b_L from eq.(B22) into eq. (B24), we get the condition for the control behaviour

$$-\left[-mL + j\frac{D^2T^2}{k+D^2T^2}L - qLT + (r_1N + r_2C)T - uNL^2 - K_L(1 - e^{-M})L + k_L(L - L^*)\right] \geq 0 \qquad \text{(B24)}$$

In eq.(B24), the desired value of cytotoxic T-cell population L^*, is given by (eq.A14), i.e.

$$L^* = \sqrt[l]{[\, s\, T^l\, U_L / (d - U_L)\,]} \qquad \text{(B25)}$$

Putting this value of L^* in the last term of eq.(B24) which is solved for the cytotoxic T-cell efficiency factor U_L there, we get:

$$U_L \geq \frac{dP^l}{sT^l + P^l} \qquad \text{(B26)}$$

where $P = L + \left(\frac{1}{k_L}\right)\left[-mL + j\frac{D^2T^2}{k+D^2T^2}L - qLT + (r_1N + r_2C)T - uNL^2 - K_L(1 - e^{-M})L\right]$

and $U_L = b_T\, g_{T_2} / r_{T_2}\, G$ [as per eq.(A11)].

Substituting these values of P and U_L in eq.(B26), we obtain the condition of the therapy weighting factor of cytotoxic T-cell, r_{T_2} ,in terms of the therapy weighting factor of temozolomide r_{T_1}, namely

$$r_{T2} \leq \frac{\left[\frac{sT^{l}+P^{l}}{dP^{l}}\right] b_T G_{T_2} - G_{T_2}^2}{G_{T_1}^2} r_{T1} \qquad \text{(B27)}$$

where, $G_{T1} = -K_T T$, $G_{T2} = -T$ [as per eq.(20)].

Rewrite eq.(B27) as $r_{T2} \leq E$ (B28)

where $E = \frac{\left[\frac{sT^{l}+P^{l}}{dP^{l}}\right] b_T G_{T_2} - G_{T_2}^2}{G_{T_1}^2} r_{T1}$ (B29)

In eq.(B29), we may note that $r_{T_1} = 1$ (sec. 2.5). The eq.(A10) and (A11) delineates the inequality requirement linking the therapy weighting factors of cytotoxic and temozolomide, viz. r_{T_1} and r_{T_2}. The eq.(B28) is the sufficient condition for administration of tumour-infiltrating lymphocyte therapy dose rate, $v_L(t) \geq 0$.

Case III: Condition for the using Interleukin injection (v_I):

We recall the desired interleukin concentration in blood I^* to enable tumour elimination [eq.(A28)]:

$$I^* = g_I U_I / (p_I L - U_I). \qquad \text{(B30)}$$

For I^* to have real value, the denominator should not be zero, i.e.

$$U_I \neq p_I L \qquad \text{(B31)}$$

The clinical efficiency factor of interleukin U_I is given in eq.(A25) which states that

$$U_I = b_L / r_{L_1} \mathrm{H}. \qquad \text{(B32)}$$

where H = [(1/ r_{L_1}) + (1 / r_{L_2})]. Substituting eq.(B32) into eq.(B31), and rearranging, we have the requirement for the value of r_{L_2} , the weighting factor of tumour infiltrating lymphocyte therapy:

$$r_{L_2} \neq \frac{p_I L r_{L_1}}{b_L - p_I L} \qquad \text{(B33)}$$

In other words, $r_{L2} \neq F$ (B34)

where $F = \frac{p_I L r_{L_1}}{b_L - p_I L}$. (B34-A)

Eq.(B34) is a *necessary condition* of administering interleukin dose, $v_I(t)$. Since this dose rate cannot be negative, we have another requirement:

$$v_I(t) \geq 0 \qquad \text{(B35)}$$

Now we consider the desired interleukin dose rate for tumour elimination v_I^* [eq.(A31)], viz.

$$v_I^* \;=\; \mu_I\, I - k_I\ (I - I^*) \qquad \text{(B36)}$$

Since the desired dose-rate $v_I^* \geq 0$, so eq.(B36) implies

$$\mu_I I - k_I(I - I^*) \geq 0 \qquad \text{(B37)}$$

Here, we now substitute the value of the desired interleukin blood level [eq.(A31)], i.e.

$$I^* \;=\; g_I\, U_I \,/\, (\, p_I\, L - U_I\,). \qquad \text{(B38)}$$

where the interleukin efficiency term U_I is given by eq.(A25), that is,

$$U_I \;=\; b_L \,/\, r_{L_1} \mathrm{H} \qquad \text{(B39)}$$

Here, H = [($1/\, r_{L_1}$) + ($1\,/\, r_{L_2}$)], as per eq.(A27). In eq.(B39), the cytotoxic T-cell activation term b_L , is obtained from eq.(A23), viz.

$$b_L = -f_L(X) - k_L(L - L^*) \qquad \text{(B40)}$$

where $f_L(X) = -mL + j\frac{D^2T^2}{k+D^2T^2}L - qLT + (r_1N + r_2C)T - uNL^2 - K_L(1 - e^{-M})L$ (B41)

Note that the cytotoxic T-cell activation term b_L can be either facilitative and positive ($b_L \geq 0$), or negative and inhibitory ($b_L \leq 0$). There are considerable experimental evidences for this bimodality [B2, B3]. For instance, tumour cell fragments and debris can cause activation of those T-cells, while such T-cells can undergo suppression if they interact with tumour cells repeatedly. Now, let us put the respective substitutions mentioned above [eq.(B38)-(B39), (B40)-(B41)] into eq. (B37), and solve for the r_{L_2} weighting factor. To write the solutions in a compact form, we let

$$G = \frac{r_{L1}}{\dfrac{\dfrac{b_L g_I}{I\left(1-\frac{\mu_I}{k_I}\right)} + b_L}{p_I L} - 1} \qquad \text{(B42)}$$

Solving for r_{L_2}, we arrive at two versions depending on whether the cytotoxic T-cell activation is facilitated or inhibited, i.e. on the b_L value:

(a) If $b_L \leq 0$ (T-cell inhibition): then one condition needs to be satisfied, viz. $r_{L_2} \leq$ G (B43)

(b) If $b_L \geq 0$ (T-cell facilitation): then two conditions need to be satisfied, viz. $r_{L_2} \geq$ G (B44)

$$\text{and } b_L \;<\; p_I\, L\, [\, 1 + (r_{L_1} / r_{L_2})\,] \qquad \text{(B45)}$$

where $b_L \;=\; U_I + v_L$ (eq. A23), indicating that b_L acts as the sum of the immunomodulative effects exerted on the cytotoxic T-cells by the interleukin efficiency factor U_I and tumour-infiltrating lymphocyte dosing v_L.

Eqs.(B34), (B43) and (B44)-(B45) are the *sufficient condition* on r_{L_2} for using interleukin dose $v_I(t)$.

.

B.2 Computation of the values of therapeutic weights r_{T_2} , r_{L_2}

From the above, we have several numerical indices, viz. the parameters named (A, B, C, D, E, F, G) defined in eqs.(B-10A), (B15-A), (B29), (B34A) and (B42), and these indices determine the values that can be taken by the tuning parameters r_{T_2} and $r_{L\,2}$ [eqs.(B11)-(B12), (B16)-B(17), (B28), (B34), (B43)-(B44)]. These numerals A-F are function of the biological and pharmacological variables associated with the tumour (*T, N, L, C, M, I)* which change temporally, thus the r_{T_2} and $r_{L\,2}$ values will change with time.

Calculating r_{T_2}:

(i) For the case when the tumour cell lysis term $b_T \leq 0$:

We have the three conditions from eqs., (B11), (B16), (B28), respectively:

$$A \leq r_{T2} < B\,; \qquad C < r_{T2} \leq D \quad \text{and} \quad r_{T2} \leq E$$

Since D = ∞ [see the line above eq.(B16)], the last two inequalities above can be combined to yield that $C < r_{T_2} \leq E$.

Now we have the two inequalities: A ≤ r_{T_2} < B, and C < r_{T_2} < E. Evidently, both these inequalities can be satisfied if we have a more stringent inequality:

[the greater value among A and C] ≤ r_{T_2} < [the smaller value among B and E] (B46)

To initiate, we can take the value of r_{T_2} to be the midway between the upper and lower bounds of r_{T_2}, these bounds respectively being the bracketed left-side and right sided expressions of eq.(B46). Thus,

r_{T_2} = ½ . [(the greater value among A and C) + (the smaller value among B and E)] (B47)

Thereby, in case r_{T_2} becomes negative, then we consider only the upper bound of r_{T_2}, so that it has a positive value, thus we take

r_{T_2} = ½ . [smaller value among B and E] (B48)

(iii) For the case when the term $b_T \geq 0$:

We consider eq.(B12), (B17), (B28), from which we similarly arrive at the inequalities

$$A \geq r_{T_2} > B, \quad \text{and} \quad C > r_{T_2} \geq E \qquad \text{(B49)}$$

Likewise, r_{T_2} = ½ . [(the greater value among B and E) + (the smaller value among A and C)] (B50)

If this gives r_{T_2} a negative value, then we delineate that

$$r_{T_2} = ½ \,.\, [\text{smaller value among A and C}] \qquad \text{(B51)}$$

If this value is also negative, then we will not select the drug dosage associated with r_{T_2}, namely $v_M\ (t)$ and $v_L\ (t)$, i.e. chemotherapy and tumour-infiltrating lymphocytes respectively. [Note that, as per eq.(A15)-(A16), the desired blood levels of chemotherapy and tumour-infiltrating lymphocytes both depend on the value of the index G therein, which in turn depend on r_{T_2} [eq.(A12)]. Thus the dosages of these two drugs are omitted (i.e. made zero), and only the other drug, interlukin-2, is administered.

Calculating $\mathbf{r_{L_2}}$:

(i) For the case that the cytotoxic T-cell effect term $b_L \le 0$:

The pertinent formulas for r_{L_2} are eqs.(B34) and (B43), respectively $r_{L_2} \neq$ F, and $r_{L_2} <$ G. Hence, we can take that

$$r_{L_2} = \text{G}-1. \qquad \text{(B52)}$$

In case if (G–1) = *F*, then we have

$$r_{L_2} = \text{G} - 2. \qquad \text{(B53)}$$

(ii) For the case when the term $b_L \ge 0$:

Attending to eq.(B34) and (B44)-(B45), one discerns that three conditions should be satisfied: $r_{L_2} \neq$ F, $r_{L_2} \ge$ G, along with $b_L < p_I\, L\, [1+(\, r_{L_1} / r_{L_2}\,)]$. We initially put

$$r_{L_2} = \text{G} + 2. \qquad \text{(B54)}$$

In case, if the aforesaid b_L inequality is not satisfied, then we put

$$r_{L_2} = \text{G} + 1. \qquad \text{(B55)}$$

However, if r_{L_2} = F, then we take that

$$r_{L_2} = \text{G} + 0.5. \qquad \text{(B56).}$$

.
.

B.3 Summary of procedures for obtaining the therapeutic weights

All the values of the tuning parameters are calculated so as to be positive.

- **Requirement for administering temozolomide dose-rate, $v_M(t)$:**

***Case I**:* If antitumour effect parameter $b_T \leq 0$:
then the antitumour drug weight tuning parameter r_{T_2} should be within the range $A \leq r_{T_2} < B$.

Case II: If $b_T > 0$: then r_{T_2} should be within the range $A \geq r_{T_2} > B$.

- **Requirement for administering tumour-infiltrating lymphocyte dose-rate $v_L(t)$:**

Case I: If $b_T \leq 0$: then r_{T_2} should be within the range $C < r_{T_2} \leq E$.

Case II: If $b_T > 0$: then r_{T_2} should be within the range $C \geq r_{T_2} > E$.

- **Requirement for administering interleukin dose-rate $v_I(t)$:**

Case I: If T-cell activation effect parameter $b_L \leq 0$:
then the T-cell drug activation parameter r_{L_2} should satisfy $r_{L_2} \leq G$ and $r_{L_2} \neq F$.

Case II: If $b_L > 0$:
then r_{L_2} should satisfy $r_{L_2} \geq G$, $r_{L_2} \neq F$, and $b_L < p_I L [1 + (r_{L_1} / r_{L_2})]$.

To sum up, that both the tuning weights r_{T_1}, $r_{L_1} = 1$ [eq.(B1)], while the values of the tuning weights r_{T_2} and r_{L_2} depend on the boundary indices A, B, C, D, F and G, whose values can determined as per eqs.(B10-A), (B10-B), (B15-A), (B29), (B34-A) and (B42), as per Table B.3 in the next page.

Table B.3 Formulation of boundary parameters that provide therapeutic weights r_{T_2} , r_{L_2}

Boundary Indices	Expression	Characterizing equation
A	$A = g_{T_1}^{\ 2}\ r_{T_1} / [\{ b_T\, g_{T_1} /1 - exp(M\gamma/k_M - M)\} - g_{T_1}^{\ 2}]$	Eq.(B10-A)
B	$B = g_{T_2}^{\ 2}\ r_{T_1} / (b_T\, g_{T_1} - g_{T_1}^{\ 2})$	Eq.(B10-B)
C	$C = b_T\, g_{T_2}/dG$	Eq.(B15-A)
E	$E = \frac{\left[\frac{sT^l + P^l}{dP^l}\right] b_T G_{T2} - G_{T2}^2}{G_{T1}^2} r_{T1}$	Eq.(B29)
F	$F = \frac{p_I L r_{L1}}{b_L - p_I L}$	Eq.(B34-A)
G	$G = \frac{1 r_{L1}}{\frac{\frac{b_L g_I}{I\left(1 - \frac{\mu_I}{k_I}\right)} + b_L}{p_I L} - 1}$	Eq.(B42)

Note that one can put r_{T_1} , $r_{L_1} = 1$ in the expressions in the middle column [see eq.(B1)]. The values of the other tuning weights r_{T_2} and r_{L_2} can be obtained by substituting the values of the boundary indices A-G (middle column) in eq.(B46)-(B56).

.
.

B.4 Submodal Protocols: One or Two Therapeutic Agents

As delineated in the earlier pages (items B.1-B.2), we can select the different dosages of the three drugs, based on the values of the therapy weights, r_{T2} and r_{L2} , which depends on the values of bounds A, B, C, D, E and F therein. We initially select all the three agents. Nevertheless, when there is violation of any of the necessary or sufficient condition/s mentioned in that section, then that specific violating drug is stopped, and the remaining two drugs continue to be administered. Thereafter, if another conditions fails, then the corresponding violating drug is omitted, we go for the remaining drug. Here we elucidate the approach for the situation necessitating the use of two drugs or of one drug, i.e. omit one or two of the three therapeutic modes (chemotherapy, immunotherapy, or cytotherapy agent), thus resulting in submodal protocols.

(a) Two drug formulation

In this method three different cases arise as three different drugs are available and two distinct drugs are used at a time. The aim is same as in three drug control, i.e., to make tumour cell population $T = 0$ in finite time. Actually, in the derivations below, we utilize the overall approach developed for the complete protocol of three drugs [Additional File 2; items A.2 & A.3). The three cases are either chemotherapy with cytotherapy, or cyto-therapy with immunotherapy, or immunotherapy with chemotherapy. The detailed elucidations follow:

Case I : Chemotherapy with Tumour-infiltrating lymphocyte therapy, $v_M(t)$ and $v_L(t)$

(A) Tumor cell compartment:

An analysis like that in eq.5 of the text, furnishes the negative bias formulation of tumour cell extinction:

$$\left(\dot{T} - \dot{T}^*\right) + k_T(T - T^*) = 0$$

This is similar to the three drug control design, and as per eq.(A14), we have the desired concentration of chemotherapy in blood (M*) and desired population of cytotoxic T-cells in blood (L*) as respectively:

$$M^* = -ln(1 - U_M)$$

$$L^* = \left(\frac{U_L s T^l}{d - U_L}\right)^{\left(\frac{1}{l}\right)}$$

(B) Cytotoxic T-cell compartment:

Following the same methodology as in eq.(A17), one arrives at the dynamic control formulation for T-cell induced tumour regression:

$\left(\dot{L} - \dot{L}^*\right) + k_L(L - L^*) = 0$

and the desired injected dose rate of tumour-infiltrating lymphocyte:

$v_L(t) = -\left(f_L(X) + k_L(L - L^*)\right)$

where $f_L(X) = -mL + j\frac{D^2T^2}{k+D^2T^2}L - qLT + (r_1N + r_2C)T - uNL^2 - K_L(1 - e^{-M})L + \frac{p_I LI}{g_I + I}$

(C) Chemotherapy compartment:

Likewise, we obtain the dynamic control formulation for chemotherapy-induced tumour regression:

$\left(\dot{M} - \dot{M}^*\right) + k_M(M - M^*) = 0$

whilst the desired injected dose rate of tumour-infiltrating lymphocyte is:

$v_M(t) = \gamma M - k_M(M - M^*)$

Case II: Tumour-infiltrating lymphocyte therapy with Interleukin, $v_L(t)$ and $v_I(t)$:

(A) Tumor cell compartment:

An aforesaid analysis furnishes the negative bias formulation of tumour cell extinction:

$\left(\dot{T} - \dot{T}^*\right) + k_T(T - T^*) = 0$

and the therapeutic efficiency factor of cytotoxic T-cell as

$$U_L = \left(\frac{1}{T}\right)[\, aT(1 - bT) - cNT - K_T(1 - e^{-M})T + k_T(T - T^*)\,] \qquad \text{(B57)}$$

with the desired population of cytotoxic T-cell in blood

$$L^* = \left(\frac{U_L sT^l}{d - U_L}\right)^{\left(\frac{1}{l}\right)}$$

This desired value of L^*, is the objective for the performance of the cytotoxic T-cell compartment. The value of U_L is obtained from eq.(B57).

(B) Cytotoxic T-cell compartment:

The expressions that we obtain is similar to the three drug control (given in main text), namely:

Therapeutic efficiency factor of interleukin $U_I = \frac{b_L}{r_{L1}\left(\frac{1}{r_{L1}}+\frac{1}{r_{L2}}\right)}$

Injected dose rate of tumour-infiltrating lymphocyte $v_L = \frac{b_L}{r_{L2}\left(\frac{1}{r_{L1}}+\frac{1}{r_{L2}}\right)}$

Desired interleukin-2 concentration in the blood: $I^* = \frac{U_I g_I}{p_I L - U_I}$

(C) Interleukin compartment:

This is reminiscent of the three drugs control design and one has

Desired injected dose-rate of interleukin: $v_I(t) = \mu_I I - k_I(I - I^*)$

***Case III*: Interleukin and chemotherapy $v_I(t)$ and $v_M(t)$:**

(A) Tumor cell compartment:

As earlier, we have the negative bias formulation of tumour cell extinction:

$$\left(\dot{T} - \dot{T}^*\right) + k_T(T - T^*) = 0$$

This is similar to the three drugs control design,

Desired blood concentration of chemotherapy agent: $M^* = -ln(1 - U_M)$

Desired population of cytotoxic T-cells: $L^* = \left(\frac{U_L s T^l}{d - U_L}\right)^{\left(\frac{1}{l}\right)}$

(B) Cytotoxic T-cell compartment:

As shown before, the dynamic control formulation for T-cell induced tumour regression:

$$\left(\dot{L} - \dot{L}^*\right) + k_L(L - L^*) = 0$$

and the therapeutic efficiency factor of interleukin is

$$U_I = -\left(-mL + j\frac{D^2T^2}{k + D^2T^2}L - qLT + (r_1 N + r_2 C)T - uNL^2 - K_L(1 - e^{-M})L + k_L(L - L^*)\right)$$

Desired interleukin-2 concentration in the blood $I^* = \frac{U_I g_I}{p_I L - U_I}$

(C) Chemotherapy compartment:

Reminiscent of the three drugs control design, one gets

Desired chemotherapy concentration in the blood $v_M(t) = \gamma M - k_M(M - M^*)$

(D) Interleukin compartment:

As before, the Desired interleukin concentration in blood $v_I(t) = \mu_I I - k_I(I - I^*)$

(b) One drug formulation

Initially we start with the Tumour cell dynamics and then with any one of the three therapies:

Case I: Chemotherapy, $v_M(t)$

In this case we need to consider successively the tumour cell dynamics and the chemotherapy input.

(A) Tumour cell compartment:

Here we have two compartments to consider: the tumour cell compartment, and its preceding compartment, the chemotherapy module (Figure 1). Following the same methodology, we see

$\left(\dot{T} - \dot{T}^*\right) + k_T(T - T^*) = 0$, and

Desired chemotherapy concentration in blood $M^* = -ln(1 - U_M)$

Here, the chemotherapy efficacy factor $U_M = -\left(\frac{1}{G_T(X)}\right)\left(f_T(X) + k_T(T - T^*)\right)$

where $G_T(X) = -k_T T$ and $f_T(X) = aT(1 - bT) - cNT - DT$.

(B) Chemotherapy compartment:

Reminiscent of the chemotherapy compartment of the three drugs control design, we have

Desired injected dose-rate of the chemotherapy drug $v_M(t) = \gamma M - k_M(M - M^*)$

Case II: Tumour-infiltrating lymphocyte therapy $v_L(t)$

In this case, we attend to the tumour cell compartment and its predecessor, the tumour infiltrating lymphocyte one (Figure 1).

(A) Tumour cell compartment:

Since we are dealing with immunomodulation, we attend to the tumour cell compartment when under the immunomodulatory terms, the dosages of tumour infiltrating lymphocyte and of interleukin, i.e. the two drug dose rates, $v_L(t)$ *and* $v_I(t)$. Thereby, we have

Desired cytotoxic T-cell population in blood $L^* = \left(\frac{U_L s T^l}{d - U_L}\right)^{\left(\frac{1}{l}\right)}$

(B) Cytotoxic T-cell compartment:

This parallels the cytotoxic T lymphocyte compartment under the effect of two drugs, viz. the dosage rates of chemotherapy and tumour-infiltrating lymphocytes, i.e. the injection terms $v_M(t)\ and\ v_L(t)$.

Desired dose-rate of tumour-infiltrating lymphocytes $v_L(t) = -\left(f_L(X) + k_L(L - L^*)\right)$

where $f_L(X) = -mL + j\frac{D^2T^2}{k+D^2T^2}L - qLT + (r_1N + r_2C)T - uNL^2 - K_L(1 - e^{-M})L + \frac{p_ILI}{g_I+I}$

Case III: Interleukin therapy, $v_I(t)$

Since the interleukin dosage module $v_I(t)$, is farthest from the tumour cell compartment, we need to consider the three serial entities: tumour cell, cytotoxic T-cell and interleukin compartments.

(A) Tumour cell compartment:

The derivation is same as the tumour cell compartment of the two drug approach, when dose-rates of tumour-infiltrating lymphocytes and interleukin, $v_L(t)\ and\ v_I(t)$, are used. Thence

Desired cytotoxic T-cell population in blood $L^* = \left(\frac{U_L s T^l}{d-U_L}\right)^{\left(\frac{1}{l}\right)}$

(B) Cytotoxic T-cell compartment:

The derivation is same as the cytotoxic T-cell compartment of two drug formulation, where one uses chemotherapy and interleukin dosage rates, $v_M(t)$ and $v_I(t)$. Thereby

Desired interleukin concentration in blood $I^* = \frac{U_I g_I}{p_I L - U_I}$

(C) Interleukin compartment:

This resembles the three drug approach, whereby

Desired dose rate of interleukin $v_I(t) = \mu_I I - k_I$

References

B1. Khalil H: 1996. *Nonlinear Systems.* Princeton: Prentice Hall; 1996.

B2. Rosenberg S, Lotze M: **Cancer immunotherapy using interleukin-2 and interleukin-2-activated lymphoytes.** *Annu. Rev Immunol.* 1986, **4:** 681–709.

B3. Gett A., Sallusto F, Lanzavecchia A., Geginat J: **The T cell fitness determined by signal strength**. *Nature Immunol*. 2003, **4(4):** 355–360.
